# Hemostatic findings of pleural fluid in dogs and the association between pleural effusions and primary hyperfibrino(geno)lysis: A cohort study of 99 dogs

**DOI:** 10.1371/journal.pone.0192371

**Published:** 2018-02-20

**Authors:** Andrea Zoia, Michele Drigo, Christine J. Piek, Paolo Simioni, Marco Caldin

**Affiliations:** 1 Department of Internal Medicine, San Marco Veterinary Clinic, Padua, Italy; 2 Department of Medicina Animale, Produzione e Salute, Padua University, Legnaro, Italy; 3 Department of Clinical Sciences of Companion Animals, Faculty of Veterinary Medicine, Utrecht University, Utrecht, The Netherlands; 4 Department of Cardiologic, Thoracic and Vascular Sciences, University of Padua Medical School, Padua, Italy; 5 Department of Clinical Pathology, Laboratorio d’Analisi Veterinarie San Marco, Padua, Italy; Duke University School of Medicine, UNITED STATES

## Abstract

The primary objective of this study was to determine if activation of coagulation and fibrinolysis occurs in canine pleural effusions. Thirty-three dogs with pleural effusions of different origin were studied. Pleural effusion fibrinogen concentrations were significantly lower, while pleural fibrin-fibrinogen degradation products (FDPs) and D-dimer concentrations were significantly higher than those in plasma (*P* < 0.001 for all comparisons). These results show that, in canine pleural fluids, there is evidence of coagulation activation and fibrinolysis. The secondary aims of the current study were to determine if primary hyperfibrinolysis ([PHF] i.e., elevated plasma FDPs with a normal D-dimer concentrations), occurs in dogs with pleural effusion, and whether the presence of a concurrent inflammatory process may have activated the hemostatic cascade, with its intrinsically linked secondary hyperfibrinolysis, masking the concurrent PHF. The previously 33 selected dogs with pleural effusion (group 1) were compared to two control groups of 33 healthy (group 2) and 33 sick dogs without pleural effusion (group 3). Serum fibrinogen, FDPs, D-dimer, C-reactive protein (CRP), fibrinogen/CRP ratio, and frequency of PHF were determined. Fibrinogen, FDPs, D-dimer and CRP concentrations in group 1 were significantly increased compared to group 2 (*P* < 0.001 for all comparisons). FDPs and CRP concentrations in group 1 were also significantly increased compared to group 3 (*P* = 0.001 and *P* < 0.001, respectively). The fibrinogen/CRP ratio was significantly decreased in group 1 compared to groups 2 and 3 (*P* < 0.001 for both comparison). The frequency of PHF was significantly higher in group 1 compared to groups 2 (*P* = 0.004), but not compared to group 3. These results support the hypothesis that PHF occurs significantly more often in dogs with pleural effusion compared to healthy dogs. Nevertheless, the decrease in the fibrinogen/CRP ratio in group 1 compared to group 3, considering the higher FDPs and similar D-dimer concentrations, would suggest that PHF is also more frequent in dogs with pleural effusion compared to sick control dogs, and that this phenomenon is hidden due to concurrent secondary hyperfibrinolysis.

## Introduction

Fibrinolysis is the process whereby stable fibrin strands are broken down by plasmin [1]. Localized fibrinolysis in response to thrombosis is necessary for the re-establishment of blood flow, and has been termed physiologic fibrinolysis [2]. Pathologic hyperfibrinolysis occurs in disease syndromes that induce increased concentrations of plasminogen activators, decreased concentrations of plasminogen inhibitors, or a combination of both [3]. Primary hyperfibrinolysis (PHF), also sometimes named primary hyperfibrinogenolysis [2,4,5], or pathologic fibrinolysis, [2] develops independently of intravascular activation of coagulation, and plasmin is formed without concomitant formation of thrombin [6]. In PHF, the production of plasmin within the general circulation overwhelms the neutralizing capacity of the antiplasmins, causing generalized fibrinogenolysis, increased production of fibrin-fibrinogen degradation products (FDPs), degradation of coagulation factors V, VIII, IX, XI [4], and degradation of any pre-existing fibrin localized in thrombi and hemostatic clots [4,6,7], potentially leading to severe bleeding [2]. Besides an excessive serum plasmin concentration, other enzymes such as serum tryptase or non-plasmic polymorphonuclear elastase have been reported as possible causes of PHF, when their serum concentration overwhelms the neutralizing capacity of the antiplasmins [8–10]. In humans, this has been associated with acute conditions, such as surgical procedures [11], shock [4], liver transplantation [12], acute leukaemia [13], or treatment with thrombolytic drugs. It can also be observed in chronic conditions such as tumours [14], chronic liver disease [15], or following peritoneovenous shunting [16–19]. Secondary or “reactive” hyperfibrinolysis, on the other hand, is a consequence of activation of coagulation causing generation of thrombin which stimulates the endothelium to produce an increased amount of tissue plasminogen activator [6]. Secondary fibrinolysis is present in virtually every patient with disseminated intravascular coagulation (DIC), as it is an appropriate response to persistent thrombin generation [2]. Because “cross-talk” between the inflammatory and hemostatic systems may be responsible for the activation of hemostasis associated with systemic inflammation [20,21], secondary or “reactive” hyperfibrinolysis, is often present in patients with inflammatory diseases.

Results of coagulation tests, more specifically FDPs and D-dimer, may help differentiate between either primary or secondary hyperfibrinolysis. During PHF, the production of FDPs is increased but the production of D-dimer is not [4]. Therefore, having elevated plasma FDPs with a normal D-dimer concentration has been suggested in human medicine as a possible indicator of PHF in the clinical setting [4,8,9]. Recently, using this criterion, we have demonstrated that dogs with ascites, above all when resulting from right-sided congestive heart failure, have abnormalities in their coagulation tests, suggesting PHF [22,23].

Pleural effusion is the pathological accumulation of fluid in the pleural space, which is classified following its pathophysiology of formation as exudate, transudate, chylous, and hemorrhagic pleural effusions [24]. Exudates form secondary to increased permeability of the pleural surface affected by inflammatory processes or neoplasia, while transudates are the result of systemic disorders altering Starling forces and form across a normal pleural surface [24,25]. Chylous effusions usually occur due to thoracic duct leak of lymph rich in triglycerides. This lymph forms from the reabsorption of the interstitial fluid of the lower extremities, abdomen (including intestinal lacteals), and thorax and is generally similar to plasma. Hemorrhagic pleural effusions occur secondary to the accumulation of blood in the pleural cavity. Consequently, all pleural effusions contain plasma or an exudate or an ultrafiltrate of plasma. Therefore, pleural effusions may contain all of the proteins/enzymes that are present in plasma, including those that participate in coagulation and fibrinolysis, as already demonstrated for ascitic fluids [26]. Fluids of virtual cavities, of which a pleural effusion is a pathological manifestation, need to be without clots to allow smooth sliding of organs over each other, and therefore clots need to be rapidly lysed. The above statement is supported by the clinical observation that pleural effusions rarely form clots *in vivo* as shown in an experiment in dogs in which the inoculation of blood or a solution containing fibrinogen and thrombin into the pleural cavity caused the activation of the coagulation system, followed by fibrinolysis [27].

Therefore, the primary objective of the study reported here was to determine if coagulation and fibrinogenolytic/fibrinolytic activity (i.e., low fibrinogen and elevated FDPs and/or D-dimer) occurs in any type of canine pleural effusion. The secondary objectives of this study were to determine if systemic clotting abnormalities suggesting PHF (i.e., elevated plasma FDPs with a normal D-dimer concentration) occurs in these dogs, and whether an inflammatory process present in these dogs may have activated the hemostatic cascade, along with its intrinsically linked secondary hyperfibrinolysis, possibly masking concurrent PHF.

## Materials and methods

### Animals

In this cohort study, the fibrinogenolytic/fibrinolytic activity of pleural effusions and the frequency of PHF in dogs with pleural effusion was compared to control dogs without pleural effusion. Group 1 included dogs with any type of pleural effusion, confirmed by thoracic radiography, ultrasonography or computer tomography, which consecutively presented to the San Marco Veterinary Clinic from September 2011 to February 2014. Dogs were only included in the study if the pleural effusion was collected and analyzed, and the pathophysiologic cause of the pleural effusion was determined. Two control groups were created. Group 2 included clinically-healthy dogs coming to the clinic for routine annual check-ups, elective surgery, blood donor health screening programs, and pre-breeding examinations, and group 3 included sick dogs without pleural effusion. Dogs in both groups 2 and 3 were chosen with the use of the electronic medical database P.O.A System-Plus 9.0^®^ from all dogs that presented to the San Marco Veterinary Clinic in the period between May 2004 and February 2014 using the following procedure. All control dogs were individually matched to group 1 dogs for age (± 6 months), sex (including neuter status), and breed. When a breed match of the same age and sex of a dog in group 1 was not found in the database, a dog with similar weight (± 5 kg) was included instead. The control dogs from groups 2 and 3 were selected as closely as possible to the admission date of the corresponding group 1 dog to reduce drift in the laboratory results. When two or more dogs fulfilled these criteria, the choice was randomly made by the computer system P.O.A System-Plus 9.0^®^. Dogs of all three groups were included in the study only if there was a complete medical record, including history and results of physical examination, complete blood count (including blood smear examination), serum biochemistry analysis, coagulation profile analysis, and urinalysis. For groups 1 and 3, the determination of a specific diagnosis for the presenting complaint was also required for inclusion in the study. Dogs of all three groups were excluded from the study if they presented with a concomitant abdominal effusion or if they had been treated with plasma, plasma derivate or anticoagulant therapy/intoxication within 30 days before study enrolment.

### Pleural effusion classification in group 1 dogs

Pleural effusions in group 1 dogs were classified according to their pathophysiology of formation. In accordance with the Starling’s law [28], transudates result from decreased colloid osmotic pressure (COP) (i.e., dogs with severe hypoproteinemia) or increased hydrostatic pressure (i.e., dogs with diseases causing venous hypertension), and exudates result from increased vascular permeability (i.e., dogs with inflammatory or neoplastic diseases directly involving the thoracic wall and/or serosal surfaces). Since COP poorly correlates with albumin in sick human patients [29], while correlating well with serum total protein in healthy dogs [30,31], hypoproteinemia was used to help identify diseases causing a decreased COP. Pleural effusion caused by blood vessel rupture was classified as a hemorrhagic effusion (i.e., dogs with iatrogenic, traumatic or spontaneous intra-thoracic bleeding). Finally, chylous effusions resulted from disruption of the thoracic duct or its tributaries [32]. Fluid triglycerides >110 mg/dL and fluid to serum triglyceride ratio > 1 were also used to identify diseases causing chylous effusion [33,34]. Diagnosis of the disease causing the pleural effusion was used as the criterion standard to establish the pathophysiology of the pleural fluid formation.

### Pleural effusion collection and measurement of parameters

A 10 mL sample of pleural effusion was collected at the time of presentation from each group 1 dog via ultrasonographic-guided thoracentesis. Fluid samples were transferred in plastic tubes with K_3_-EDTA for the determination of haematocrit and total nucleated cell count, and in plain glass tubes for the determination of total protein with an automated chemistry analyzer (Olympus AU 2700, Olympus Diagnostics, Hamburg, Germany). A board certified clinical pathologist performed the cytology examinations on all fluid samples. Fluid samples were also transferred in a 3.5 mL plastic tubes with 3.2% sodium citrate (final ratio of volume of anticoagulant to volume of blood, 1:9) (3.2% sodium citrate Vacuette^®^ 3.5 mL, Grenier Bio-One, Kremsmünster, Austria) for the measurement of coagulation variables (see next paragraph).

### Plasma and pleural effusion coagulation parameter analysis

Coagulation profile analysis was performed for all dogs and included the determination of fibrinogen, FDPs, and D-dimer. A venous blood sample was taken from the cephalic (for medium/large-size dogs) or jugular (for small size-dogs) veins for routine laboratory analysis. In dogs of group 1, venous blood samples were taken at the same time (± 2 hours) as the pleural effusion samples were collected. A 3.5 mL aliquot of this blood was transferred in a plastic tube with 3.2% sodium citrate with a final ratio of volume of anticoagulant to volume of blood of 1:9 (3.2% sodium citrate Vacuette^®^ 3.5 mL, Grenier Bio-One, Kremsmünster, Austria) for the measurement of coagulation parameters. Tubes with sodium citrate were centrifuged at 1,950 *g* for 5 min, plasma was harvested, and coagulation profile analysis was performed within 1 hour after blood sample collection. Pleural effusion and plasma fibrinogen concentrations were determined via quantitative assays (STA Fibrinogen, Diagnostica Stago, Asnières sur Seine, France), with an automated analyzer (STA-R Evolution, Diagnostica Stago, Roche, Bäsel, Switzerland), The detection limit for fibrinogen concentration was 60 mg/dL; for statistical analysis values below this concentration were entered into the data sheet as 59 mg/dL. Pleural effusion and plasma concentrations of FDPs were measured with an immunoturbidimetric quantitative assay (STA—Liatest^**®**^ FDP, Diagnostica Stago, Asnières sur Seine, France), with an automated analyzer (STA-R Evolution, Diagnostica Stago, Roche, Bäsel, Switzerland). Pleural effusion and plasma D-dimer concentrations were determined via a validated [35], immunoturbidimetric quantitative assay (Tina-quant D-Dimer, Roche Diagnostic GmbH, Mannheim, Germany), with an automated analyzer (Olympus AU 2700, Olympus Diagnostics, Hamburg, Germany).

All collection procedures were performed solely for the dog’s benefit and for standard diagnostic and monitoring purposes. Previous informed written consent was obtained from all dog owners. Anaesthesia, euthanasia, or any kind of animal sacrifice were not required for any part of the study. All the procedures performed complied with the European legislation **“**on the protection of animals used for scientific purposes” (Directive 2010/63/EU) and with the ethical requirement of the Italian law (Decreto Legislativo 04/03/2014, n. 26). Accordingly, this type of study did not require an authorization or an ID protocol number.

### Primary hyperfibrinogenolysis

Primary hyperfibrinogenolysis was defined as a discordant result between changes in plasma FDPs and D-dimer concentrations, with FDPs above the reference interval (reference interval, < 5 μg/mL) and D-dimer within the reference interval (reference interval 0.01 to 0.34 μg/mL).

### C-reactive protein

The serum C-reactive protein (CRP) concentration was measured with an automated analyzer (Olympus AU 2700, Olympus Diagnostics, Hamburg, Germany), using an immunoturbidimetric assay validated in humans (CRP OSR6147, Olympus Life and Material Science Europe GmbH, Lismeehan, O’Callaghan’s Mills, Ireland), that showed a good correlation (r = 0.98) [36] with those of a previously validated canine-specific ELISA (Tridelta Phase range canine CRP kit, Tridelta Development Ltd., Brey, Ireland).

### Statistical analysis

Kolmogorov-Smirnov test was used to assess if data were normally distributed. In group 1 only (dogs with pleural effusion), fibrinogen, FDPs, and D-dimer concentrations were compared between pleural effusion and venous blood via the Wilcoxon signed ranks test. Serum fibrinogen, plasma FDPs, plasma D-dimer, serum CRP concentrations, and the serum fibrinogen/CRP ratio were then compared between group 1, group 2 (healthy dogs), and group 3 (sick dogs without pleural effusion) via ANOVA (fibrinogen concentrations) followed by Tamhane post-hoc test or via Kruskal-Wallis tests (D-dimer, FDPs, CRP concentrations, and fibrinogen/CRP ratio) followed by a Mann-Whitney test. The Fisher's exact test was used to assess differences among and between the three groups in frequency of PHF as previously defined (i.e., elevated plasma concentration of FDPs along with a normal plasma D-dimer concentration).

For all statistical analyses, values of *P* < 0.05 were considered significant.

## Results

### Animals

During the study period, 156 dogs with any type of pleural effusion, as confirmed by thoracic radiography, ultrasonography or computer tomography, presented to the clinic. In 59 of these dogs, pleural effusion and venous blood samples were collected at presentation. Twenty-six dogs (nine due to rodenticide exposure, 14 with concurrent abdominal effusion, and three due to lack of identification of the underlying cause for the pleural effusion formation) were excluded from further analysis. The remaining 33 dogs entered the study in group 1. Eighteen dogs were male (15 sexually intact and three neutered) and 15 female (11 sexually intact and four spayed). There were five mongrel dogs, three German shepherd dogs and three Labrador retrievers. Each of the following breeds was represented by two dogs: boxer, Deutsch kurzhaar, greyhound, pug, Rottweiler, and West Highland white terrier. The remaining nine dogs were of other breeds. One dog had pleural effusion due to decreased COP, four due to increased hydrostatic pressure, 20 due to increased vascular permeability, four due to chylous effusion, and four due to haemorrhage. The underlying diseases leading to pleural effusion formation are summarized in Table 1.

**Table 1:** Causes of pleural effusions formation in the 33 dogs of group 1.

Group 1 (n = 33)				
↓ COP transudates	↑ HP transudates	Exudates	Chylous PE	Hemorrhagic PE
(n = 1)	(n = 4)	(n = 20)	(n = 4)	(n = 4)
• PLE (1)	• Neoplasia non directly involving the pleural surfaces (3) • Cardiogenic (1)	• Pyothorax (11) • Neoplasia directly involving the pleural surfaces (8) • Non septic PE (1)	• Idiopathic (3) • Cardiogenic (1)	• Malignancies (4)

↓ COP, decreased colloid osmotic pressure; ↑ HP, increased hydrostatic pressure; PLE, protein losing enteropathy; PE, pleural effusion; the number of observations is reported in brackets.

Group 2 (clinically healthy dogs) was 100% matched for sex and neutering status to group 1. Group 2 was 76% breed matched to group 1; the breed match was incomplete in 8 out of 33 dogs.

Group 3 (sick dogs without pleural effusion) was 100% matched for sex and neutered status to group 1. Group 3 was 91% breed matched to group 1; breed match was incomplete in 3 out of 33 dogs. Causes of sickness included gastrointestinal disorders (n = 6), brain diseases (5), endocrinopathies (4), sepsis and infectious diseases (5), neoplastic diseases (2), compression of the spinal cord by intervertebral disks (2), protein losing nephropathy (2), and other causes (6).

There was no statistical difference regarding age between groups 1 (7.36 ± 4.13 years; range, 0.33–16.42 years), group 2 (7.05 ± 4.10 years; range, 0.33–14.75 years) and group 3 (7.30 ± 4.06 years; range, 0.33–16.42 years; F = 0.053; *P* = 0.95).

### Pleural effusion and plasma coagulation parameters analysis in dogs of group 1

**Fig 1:**
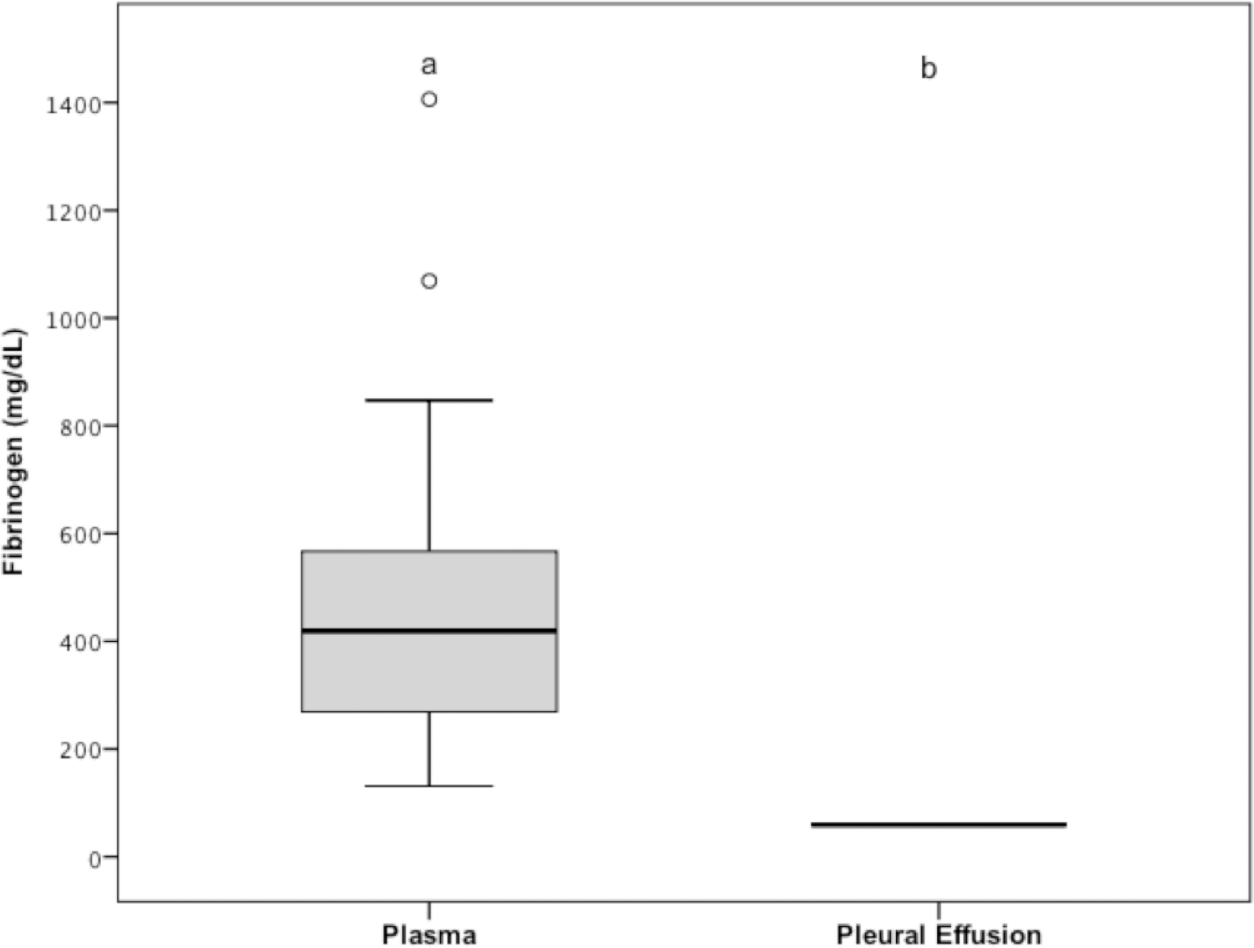
Tukey boxplots of plasma and pleural effusion fibrinogen concentrations of the 33 dogs of group 1. ^a,b^Data distributions with different letters are significantly different (*P* < 0.001). The bottom and top of the box are the 1^st^ and 3^rd^ quartiles; the median is the band inside the box. Whiskers correspond to the lowest datum still within 1.5 interquartile range of the lower quartile, and the highest datum still within 1.5 interquartile range of the upper quartile. Circles and stars are outlier and extreme outlier values (more than 1.5 and 3 interquartile range away from the closest end of the box, respectively).

Pleural effusion fibrinogen concentrations (median = 59 mg/dL; range: 59–59) were significantly lower (*P* < 0.001) than those in the plasma (median = 419 mg/dL; range: 131–1406; reference interval: 152–284 mg/dL; Fig 1), and they were below the instrument detection limit (i.e., < 60 mg/dL) in all 33 dogs. When the pleural effusion fibrinogen concentration of each dog was compared to its matching plasma value, the pleural effusion fibrinogen concentration was lower than the plasma sample in all dogs.

**Fig 2:**
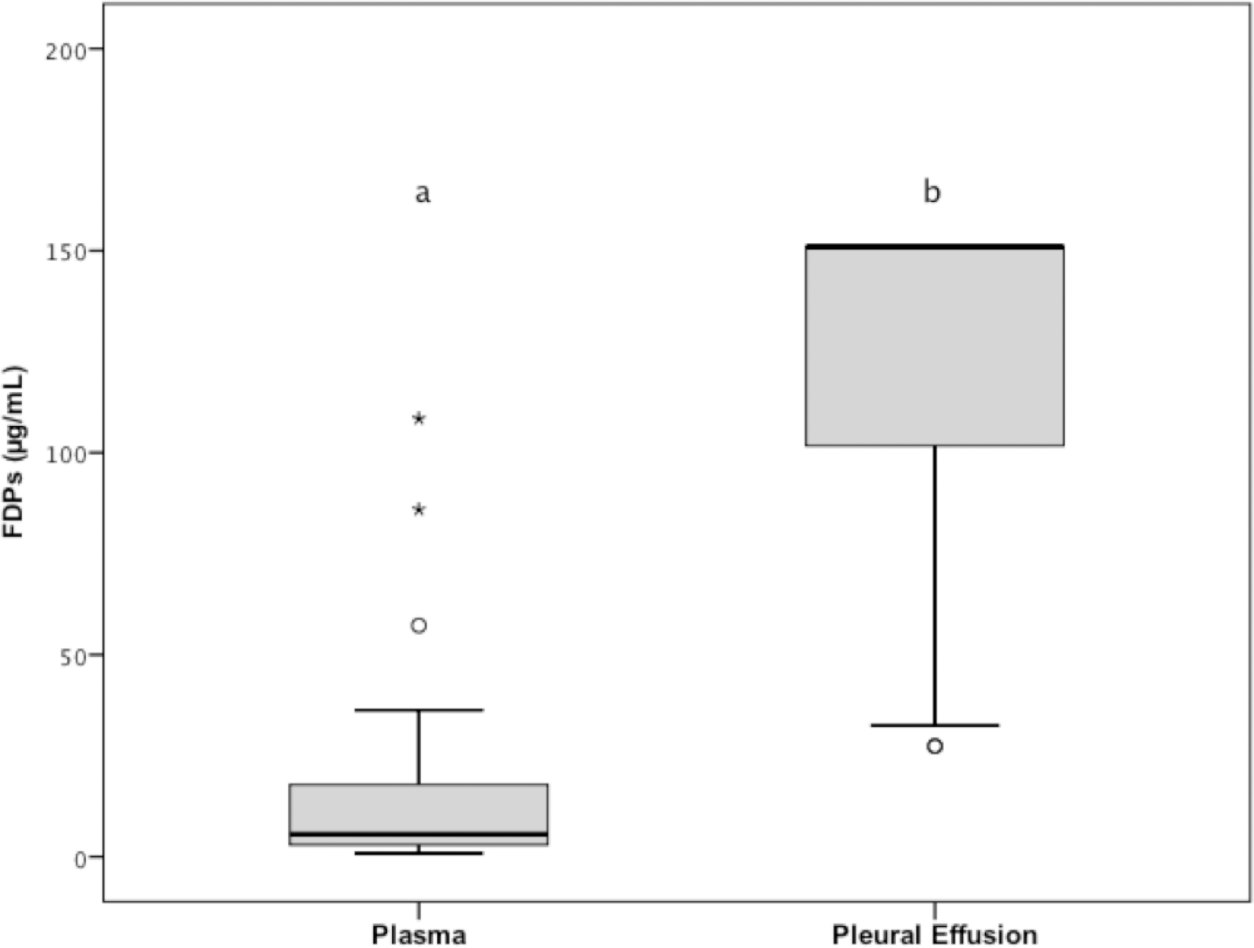
Tukey boxplots of plasma and pleural effusion quantitative FDPs concentrations of the 33 dogs of group 1. ^a,b^Data distributions with different letters are significantly different (*P* < 0.001). See Fig 1 for remainder of key. FDPs, Fibrin-fibrinogen degradation products.

The quantitative FDP concentrations in the pleural effusion (median = 151 mg/dL; range: 0.69–151) were significantly (*P* < 0.001) higher than plasma quantitative FDP concentrations (median = 5.55 mg/dL; range: 0.83–108.47; Fig 2), and they were higher than the plasma reference value (< 5 μg/mL) in all 33 pleural effusions. When the pleural effusion of quantitative FDPs concentration of each dog was compared to its matching plasma value, the pleural effusion FDPs concentration was lower than the plasma sample in all dogs.

**Fig 3:**
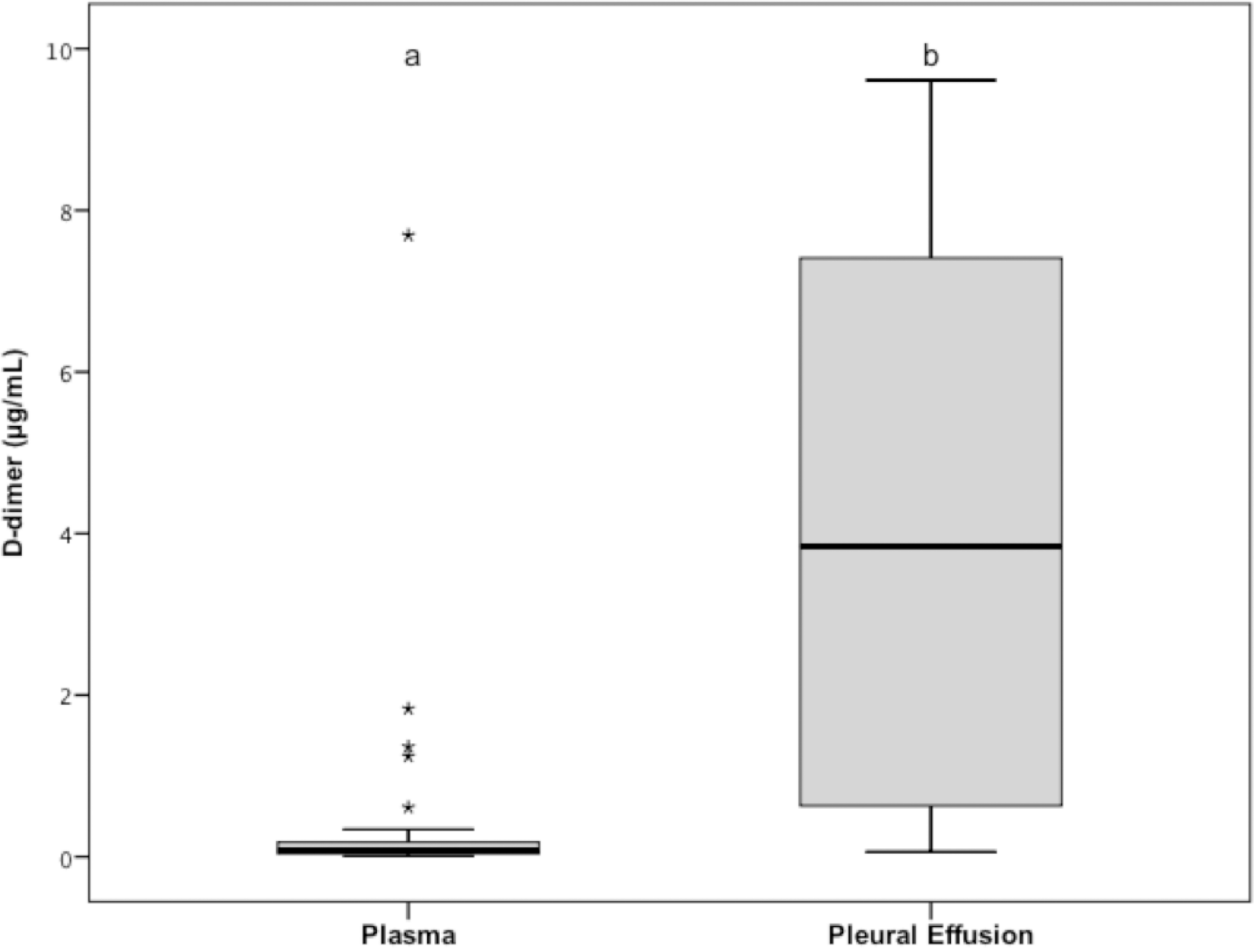
Tukey boxplots of plasma and pleural effusion D-dimer concentrations of the 33 dogs of group 1. ^a,b^Data distributions with different letters are significantly different (*P* < 0.001). See Fig 1 for remainder of key.

Pleural effusion D-dimer concentrations were significantly (*P* < 0.001) higher (median: 3.84 μg/mL; range: 0.05–9.61) than they were in the plasma (median: 0.07 μg/mL; range: 0.01–7.69; Fig 3), and they were higher than the plasma reference interval (0.01–0.34 μg/mL) in 28 out of the 33 pleural effusions. When the pleural effusion D-dimer concentration of each dog was compared to its matching plasma value, the pleural effusion D-dimer concentration was higher than the plasma sample concentration in 32 cases and lower in one case (i.e., in a hemorrhagic pleural effusion).

In conclusion, all the pleural effusions had non-measurable fibrinogen concentrations and all the 33 pleural effusions had at least one fibrin/fibrinogenolytic marker above the reference interval of what is normal for a plasma sample.

### Fibrinogen, FDPs, D-dimer, frequency of PHF, CRP, and fibrinogen/CRP ratio

**Fig 4:**
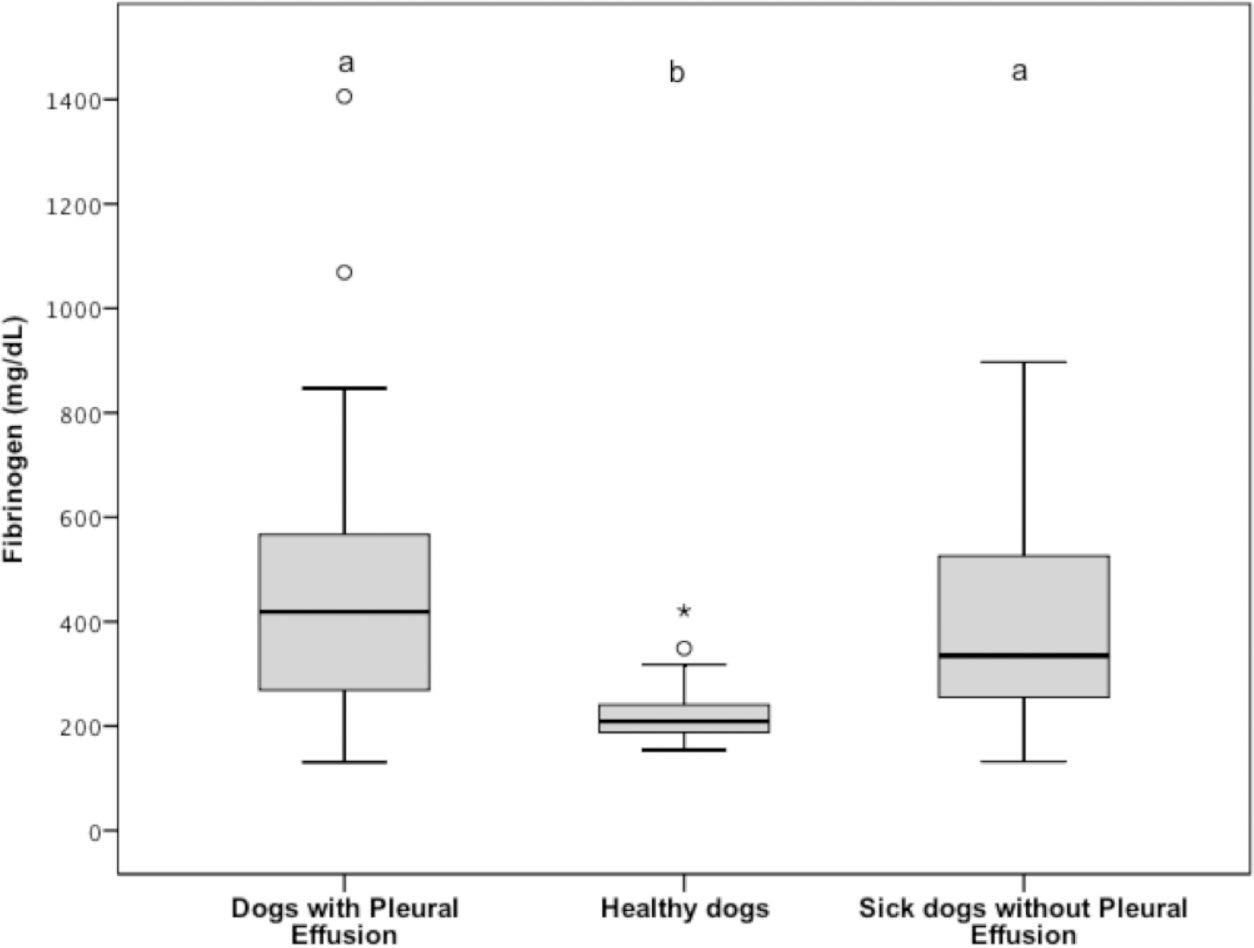
Tukey boxplots of plasma fibrinogen concentrations from dogs with pleural effusion (n = 33), healthy dogs (33), and sick dogs without pleural effusion (33). ^a,b^Data distributions with different letters are significantly different (*P* < 0.001 for both comparisons). See Fig 1 for remainder of key.

Plasma fibrinogen concentrations (reference interval, 152 to 284 mg/dL) were significantly (*P* < 0.001) higher in group 1 (mean ± SD, 469.21 ± 289.88 mg/dL; 95% CI, 366.43 to 572.00 mg/dL) versus group 2 (mean ± SD, 223.52 ± 58.55 mg/dL; 95% CI, 202.75 to 244.28 mg/dL), while they were not significantly different (*P* = 0.559) between group 1 and group 3 (mean ± SD, 395.79 ± 204.21 mg/dL; 95% CI, 323.28 to 468.20 mg/dL). Plasma fibrinogen concentrations were also significantly (*P* < 0.001) lower for group 2 versus group 3; (Fig 4).

**Fig 5:**
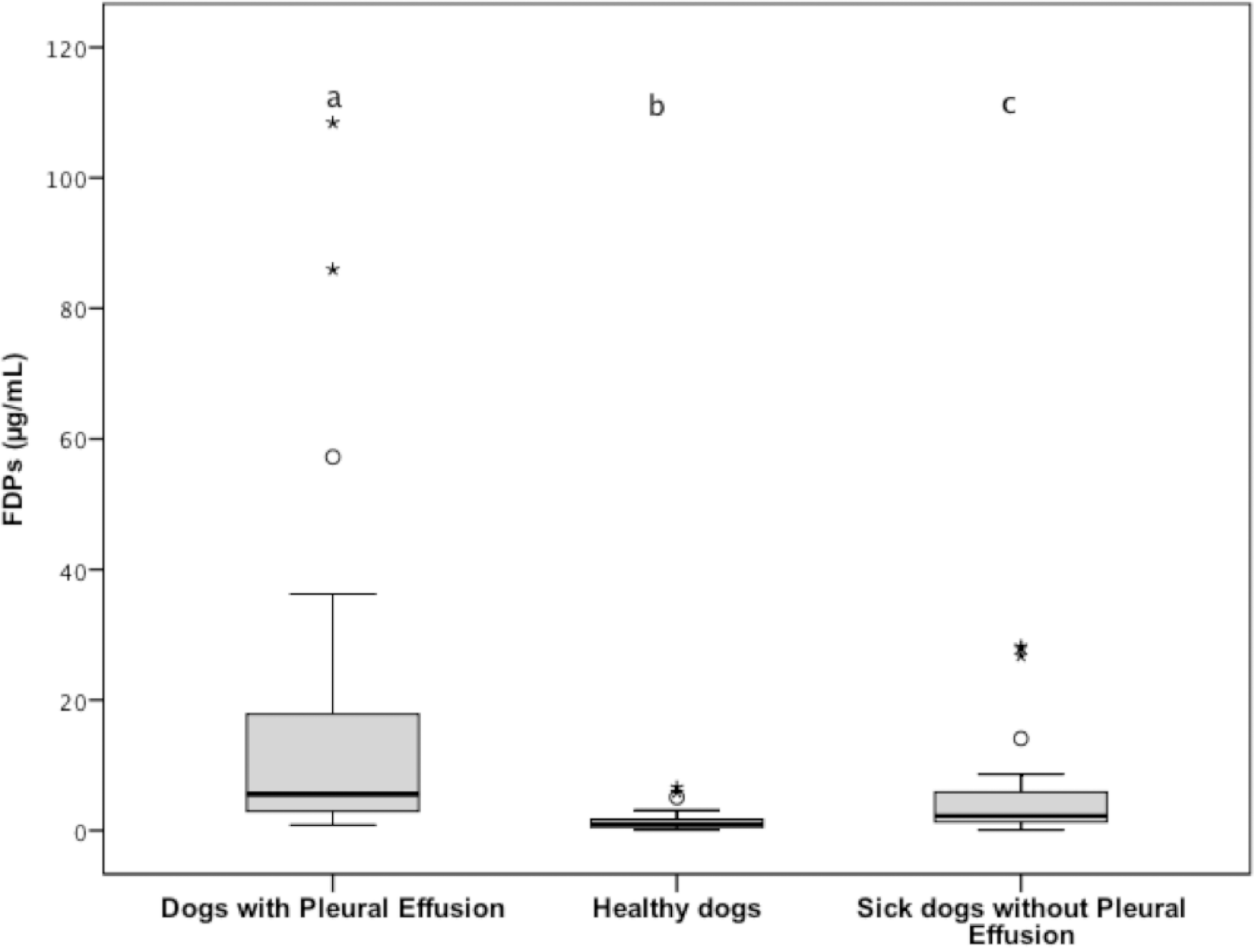
Tukey boxplots of plasma FDP concentrations from dogs with pleural effusion (n = 33), healthy dogs (33), and sick dogs without pleural effusion (33). ^a,b,c^Data distributions with different letters are significantly different (*P* < 0.001 for “a,b” comparison; *P* = 0.001 for “a,c” and “b,c” comparisons). See Fig 1 for remainder of key.

Plasma concentrations of FDPs (reference interval, < 5 μg/mL) were significantly higher (*P* < 0.001) for group 1 (median, 5.55 μg/mL; range, 0.83 to 108.47 μg/mL) versus groups 2 (median, 0.97 μg/mL; range, 0.10 to 6.61 μg/mL) and they were also significantly higher (*P* = 0.001) versus group 3 (median, 2.24 μg/mL; range, 0.10 to 28.21 μg/mL). Plasma concentrations of FDPs were also significantly (*P* = 0.001) lower for group 2 versus group 3; (Fig 5). Eighteen dogs in group 1 had plasma concentrations of FDPs above reference interval.

**Fig 6:**
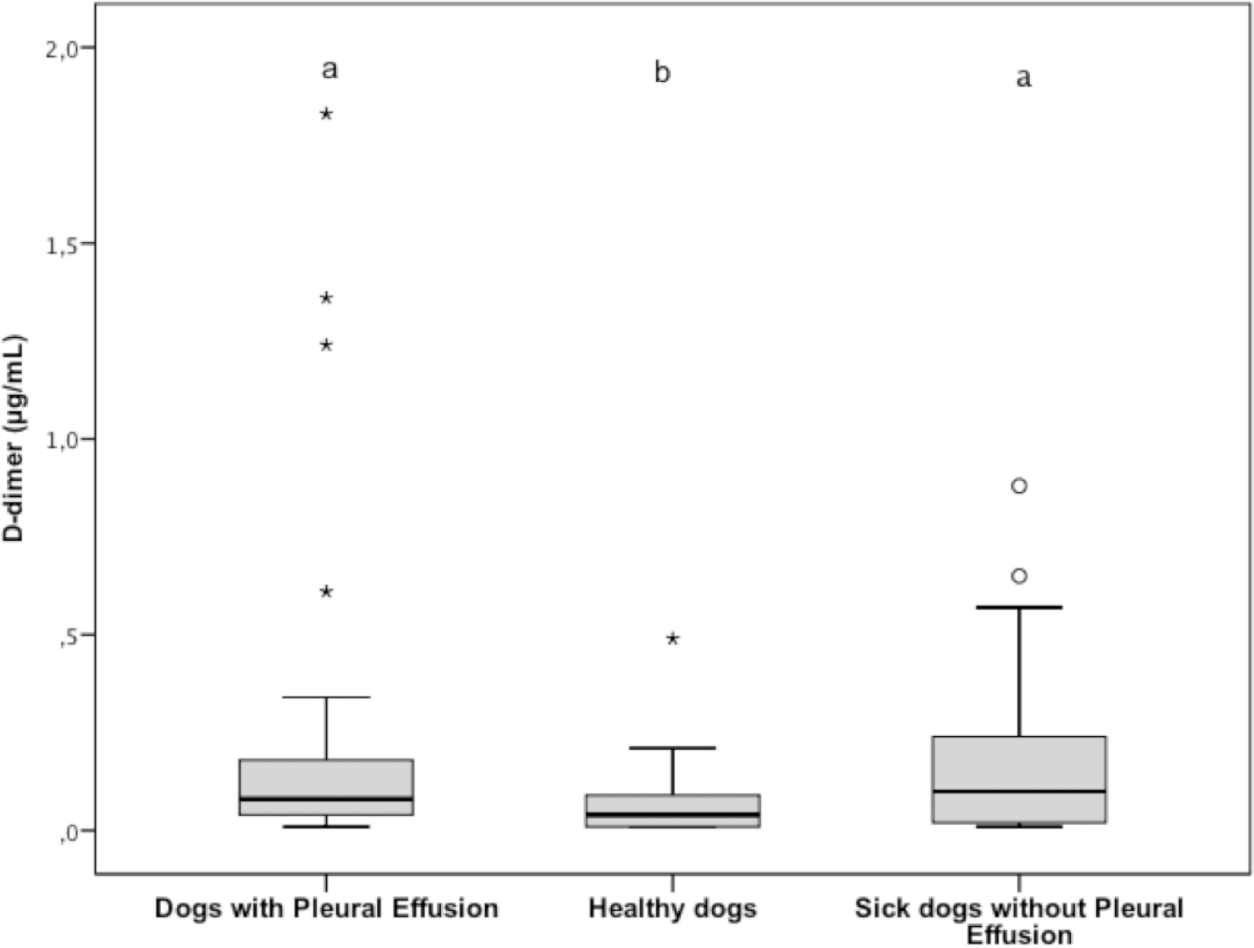
Tukey boxplots of plasma D-dimer concentrations from dogs with pleural effusion (n = 33), healthy dogs (33), and sick dogs without ascites (33). ^a,b^Data distributions with different letters are significantly different (*P* < 0.001 for both comparisons). In the graph, in group 1, it is missing an extreme outlier with a value of 7.69. See Fig 1 for remainder of key.

Plasma D-dimer concentrations (reference interval 0.01 to 0.34 μg/mL) were significantly higher (*P* < 0.001) for group 1 (median, 0.07 μg/mL; range, 0.01 to 7.69 μg/mL) versus group 2 (median, 0.04 μg/mL; range, 0.01 to 0.49 μg/mL; *P* < 0.001), while they were not significantly different (*P* = 0.964) between group 1 and group 3 (median, 0.10 μg/mL; range, 0.01 to 0.88 μg/mL). Plasma D-dimer concentrations were also significantly (*P* < 0.001) lower for group 2 versus group 3; (Fig 6). Five dogs in group 1 had plasma D-dimer concentrations above reference interval. Primary hyperfibrinogenolysis (i.e., elevated plasma concentration of FDPs with a normal plasma D-dimer concentration) was detected for 13 (39.4%) dogs in group 1, two (6.1%) dogs in group 2, and eight (24.2%) dogs in group 3. Frequency of PHF was significantly (*P* = 0.002) higher for group 1 versus groups 2. There were no significant differences in frequency of PHF between group 1 and group 3 (*P* = 0.29) and between group 2 and group 3 (*P* = 0.08). Of the 13 dogs in group 1 with PHF, one had pleural effusion due to increased hydrostatic pressure, nine due to increased vascular permeability, two due to chylous effusion, and two due to haemorrhage. The only dog included in the study with a transudative pleural effusion due to decreased COP did not have PHF. Finally, of the 13 dogs in group 1 with PHF, one had concurrent hypofibrinogenemia. None of the 11 dogs in groups 2 or 3 with PHF had hypofibrinogenemia.

**Fig 7:**
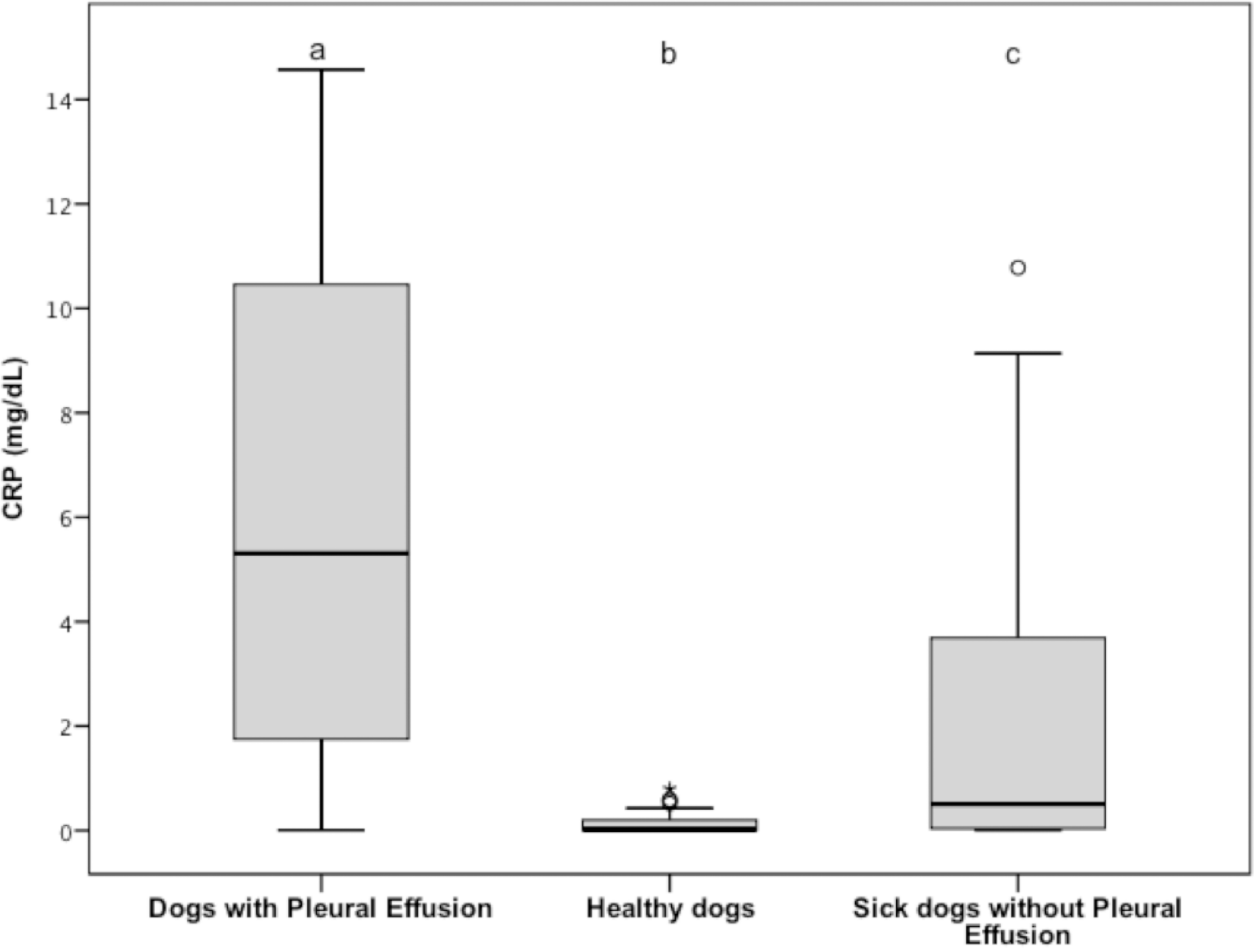
Tukey boxplots of plasma CRP concentrations from dogs with pleural effusion (n = 33), healthy dogs (33), and sick dogs without ascites (33). ^a,b,c^Data distributions with different letters are significantly different (*P* < 0.001 for all comparisons). See Fig 1 for remainder of key. CRP, C-reactive protein.

Serum CRP concentration (reference interval, 0.01 to 0.22 mg/dL) was significantly increased in group 1 (median, 5.31 mg/dL; range, 0.01 to 14.57 mg/mL) compared to group 2 (median, 0.02 mg/dL; range, 0.01 to 0.08 mg/mL) and 3 (median, 0.51 mg/dL; range, 0.01 to 10.78 mg/mL) (*P* < 0.001 for both comparison). Serum CRP concentration was also significantly lower for group 2 versus group 3 (*P* < 0.001); (Fig 7).

**Fig 8:**
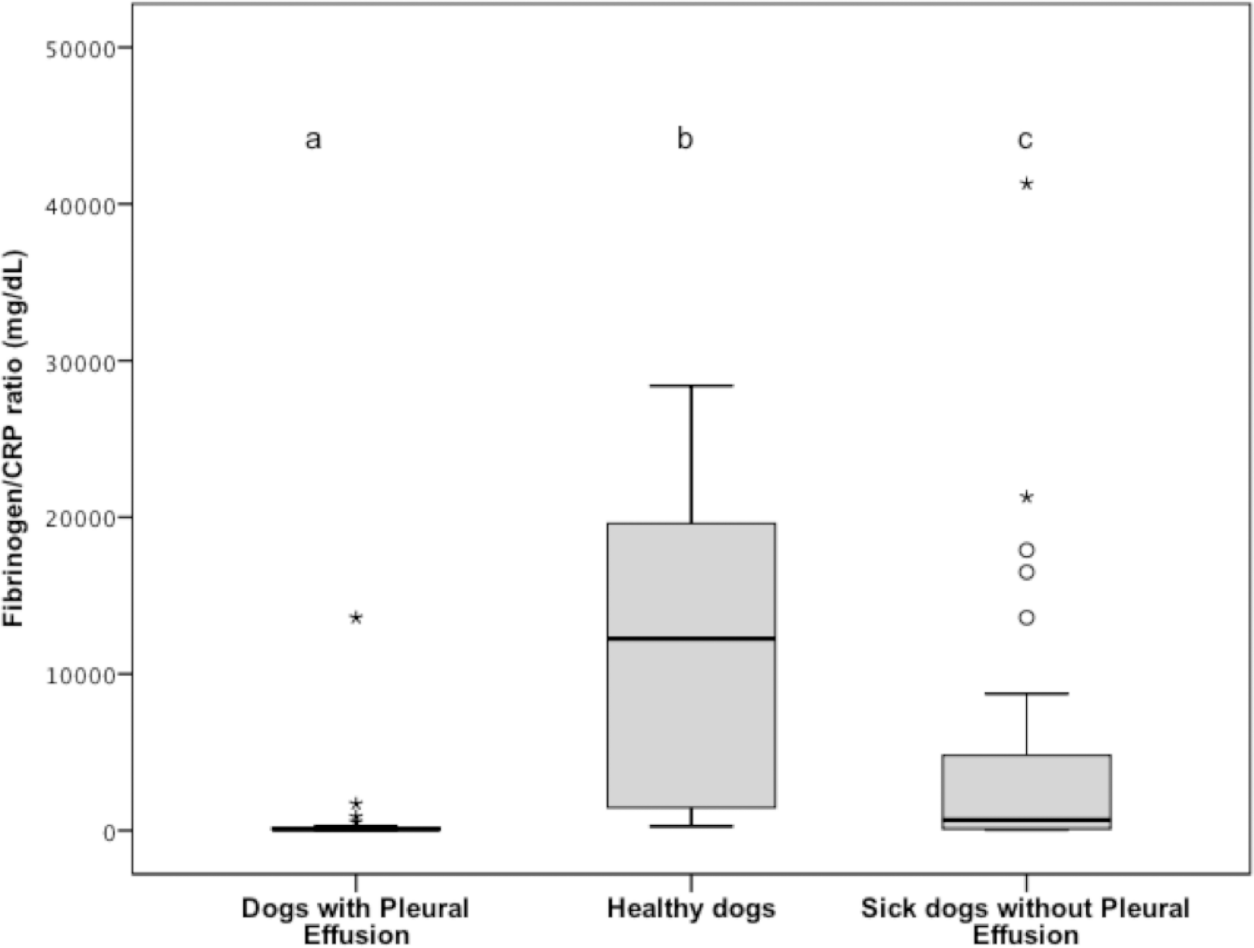
Tukey boxplots of plasma fibrinogen/CRP ratio from dogs with pleural effusion (n = 33), healthy dogs (33), and sick dogs without ascites (33). ^a,b,c^Data distributions with different letters are significantly different (*P* < 0.001 for all comparisons). See Fig 1 for remainder of key. CRP, C-reactive protein.

Serum fibrinogen/CRP ratio was significantly decreased in group 1 (median, 82 mg/dL; range, 21 to 13600 mg/mL) compared to group 2 (median, 12250 mg/dL; range, 281 to 28400 mg/mL) and 3 (median, 678 mg/dL; range, 50 to 41300 mg/mL) (*P* < 0.001 for both comparison). Serum fibrinogen/CRP ratio was also significantly lower for group 2 versus group 3 (*P* < 0.001); (Fig 8).

## Discussion

The primary aim of this cohort study was to investigate whether coagulation and fibrinogenolytic/fibrinolytic activity occurs in any type of canine pleural effusion. The results of the current study show that a) in the pleural effusion of dogs, there is evidence of coagulation activation and fibrinolysis in every case, and b) this phenomenon occurs independently of the underlying mechanism that leads to pleural effusion formation.

The first statement is supported by the finding that fibrinogen concentrations in dogs are undetectable and significantly lower in the pleural effusion, in comparison to plasma, while FDPs and D-dimer pleural fluid concentrations are significantly higher than those in the plasma. Taken together, these results would suggest that fibrinogen, upon entrance into the pleural cavity, is converted into cross-linked fibrin and then lysed to form FDPs and D-dimer, or that some fibrinogen, fibrin monomer or polymers may get lysed even before being transformed in cross-linked fibrin in FDPs. This latter hypothesis would explain the few pleural effusions with increased FDPs but with D-dimer concentrations lower than the plasma reference interval or lower compared to their corresponding plasma value. Similar conclusions have been reached by experiments in a canine model [27,37] and in the human clinical setting [38–40].

To assess if the fibrinogenolytic/fibrinolytic activity of the pleural fluid is independent of the type of pleural effusion, we included in this study effusions formed secondary to five different pathophysiological mechanisms of fluid formation (i.e., transudates due to increased hydrostatic pressure, transudates due to decreased COP, exudates, chylous and hemorrhagic pleural effusions). In the 33 included cases, the pleural fluid fibrinogen concentrations were lower than their corresponding plasma concentrations, and in all cases at least one of the fibrin/fibrinogenolytic markers in the pleural effusions were above the reference interval of what is considered normal in a plasma sample and higher than their corresponding plasma values. These findings confirm that the fibrinogenolytic/fibrinolytic activity of pleural effusion is independent of the underlying mechanism of its formation. Therefore, pleural effusions have inherent fibrin/fibrinogenolytic activity, as has been shown in the case for human [26,41,42], and ascitic equine fluid [43]. In addition, in a recent study on dogs with ascites formed secondary to different pathophysiological mechanisms, we found that also canine abdominal effusions have the same inherent fibrin/fibrinogenolytic activity [44]. The hypothesis that pleural fluid is inherently fibrinolytic is also supported not only by the clinical observation that pleural effusions rarely form clots *in vivo*, but also by several studies. The first study on the topic was conducted in 1916 and showed that the inoculation of blood or of a solution containing fibrinogen and thrombin into the pleural cavity of dogs caused the activation of the coagulation system followed by rapid fibrinogenolysis/fibrinolysis, allowing the inoculated blood to remain fluid to a large extent [27]. These initial findings were later confirmed by another experimental study in dogs [37], and by studies in humans with hemothorax [45,46], and by other studies including patients with different types of pleural effusion [47,48]. The activation of plasminogen in the pleural effusion, responsible for the documented fibrinogenolysis/fibrinolysis, may be caused by the presence of tissue plasminogen activator, urinary plasminogen activator, both enzymes, or other fibrinolytic enzymes [46,48], which can be released (from a preformed storage pool) or leak into the pleural fluid (following the damage induced by the disease causing the pleural effusion) from the mesothelial and submesothelial endothelium, inflammatory or neoplastic cells.

The secondary aim of this cohort study was to determine if systemic clotting abnormalities suggesting PHF (i.e., elevated plasma FDPs with a normal D-dimer concentration) occur in dogs with pleural effusion, and whether a possible inflammatory process present in these dogs may have activated the hemostatic cascade, along with its intrinsically linked secondary hyperfibrinolysis, possibly masking this phenomenon. The results show that a) PHF, according to our definition, occurs significantly more often in dogs with pleural effusion compared to healthy dogs, and b) although there was also a trend for increased PHF in dogs with pleural effusion compared to sick dogs, this difference did not reach significance. Nevertheless, the plasma fibrinogen concentration in dogs with pleural effusion was similar to sick control dogs (Fig 4), despite a significantly increased serum CRP concentration in comparison to these dogs (Fig 6). The resulting significant decrease in the fibrinogen/CRP ratio in dogs with pleural effusion compared to the sick control group, in the face of higher FDPs concentration and a similar D-dimer concentration, would suggest that PHF is also more frequent in dogs with pleural effusion compared to sick control dogs. Therefore, similarly to dogs with ascites [23], dogs with pleural effusion are at increased risk of PHF, which is the cause of the relative (in comparison to their inflammatory state) decreased fibrinogen concentration observed in these dogs.

In a recent study on dogs with ascites, we found that PHF occurred with different frequency in all dogs with ascites. Moreover, we found that PHF was statistically more frequent in dogs with ascites secondary to an increase in hydrostatic pressure compared to dogs in which the ascites formed secondary to another pathophysiological method. In addition, dogs with ascites secondary to an increase in hydrostatic pressure were the only dogs to have a statistically more frequent PHF in comparison to both healthy and sick dogs without ascites [23]. The results of this study showed that PHF also occurred with different frequency in all dogs with pleural effusion, with the exception of the dogs in which pleural effusion formed secondary to decreased colloid osmotic pressure. Due to the small number of dogs with pleural effusion included in this study, statistical analysis to assess if different types of pleural effusion were associated more frequently with PHF was not carried out. Furthermore, our search in the human medical literature found no studies evaluating this issue.

It is interesting to note that two (6.1%) of the healthy dogs had findings consistent with a diagnosis of PHF, but all of them only had mild elevations in FDPs, with normal plasma fibrinogen concentrations. Similar findings have been found in a recent study in a different healthy canine population [23]. Therefore, it is possible that the definition we adopted for PHF is not 100% specific for diagnosing PHF and that more stringent criteria might be required. However, it is also possible that some of our healthy animals had occult/subclinical disease resulting in PHF or that low grade PHF could be a physiological feature detectable with our definition of PHF.

In the normal state, the pleural space is a dynamic compartment in which fluids, electrolytes and proteins are continuously exchanged, with the net direction of this flux being from the parietal pleural capillaries, through the pleural space and into the visceral pleural capillaries and lymphatics. In the pathological state, pleural fluid accumulates within the thoracic cavity when more fluid enters the pleural space than is removed [49,50]. Therefore, pleural fluid, which has traditionally been regarded by physicians as an inert fluid, contains all of the proteins/enzymes that are present in plasma, including those that participate in coagulation and fibrinolysis [39]. Plasma coagulation-relevant proteins in pleural fluids are no longer contained in their natural environment (i.e. blood vessels), but are in a relatively acellular environment (i.e. the pleural space). They are also not exposed to the vascular endothelium where, according to the cell-based model of hemostasis, in which tissue factor-bearing cells and platelets are necessary for the three steps (initiation, amplification and propagation) of hemostasis [51], their actions would no longer be well-regulated. This lack of regulation could result in the formation of a fluid with an inherently increased fibrinogen/fibrinolytic activity, as already demonstrated to be the case for ascitic fluid [23,26,41,42]. The increased frequency of PHF documented in this study in dogs with pleural effusion, compared to healthy dogs or sick dogs without pleural effusion, might possibly be explained by the fibrinolytic activity of all types of pleural effusion, which upon re-entering the systemic circulation via the thoracic duct and the pulmonary veins might possibly contribute to the systemic hyperfibrinolytic state found in 39.4% of our dogs with pleural effusion.

One limitation of the current study is that the pleural fibrinogen, FDP, and D-dimer concentrations were measured using kits that had been validated only in canine plasma samples and not in canine pleural effusion samples. Nevertheless, several studies in humans and horses also used plasma validated kits for fibrinogen, FDPs, and D-dimer for the determination of these analytes in intracavitary effusions, without apparent problems [41–43]. A second limitation of the current study is that PHF was diagnosed indirectly, by measuring FDPs and D-dimer [4,8,9], while fibrinolytic enzymes responsible for PHF such as plasmin, serum tryptase, or non-plasmic polymorphonuclear elastase were not measured [2,8–10], neither in the pleural effusion nor in the plasma. Therefore, it may be argued that the increased FDP plasma concentrations are the result of their reabsorption from the pleural fluid rather than de novo formation in the plasma secondary to a systemic PHF. However, despite the possibility that some the plasma FDPs in dogs with pleural effusion originate from the pleural effusion, this does not explain the discrepancy found between their plasma FDPs and plasma D-dimer. In fact, if plasma FDP and D-dimer concentrations were derived only from the pleural fluid, we would expect both of them to be increased in a similar proportion since, as demonstrated in this study, both pleural effusion FDPs and D-dimer concentrations were almost always higher than the plasma reference interval. Conversely, plasma FDPs were increased in 18 of these dogs, while plasma D-dimer was increased in only five cases.

## Conclusions

In summary, we have shown that pleural effusions have fibrinolytic activity independent of the underlying mechanism of intra-thoracic fluid accumulation, and we also demonstrated that almost 40% of these dogs have systemic coagulative alterations suggesting PHF. The true frequency of PHF in dogs with pleural effusion could have been underestimated in this study due to the concurrent presence of secondary hyperfibrinolysis caused by the inflammatory disease underlying the cause of pleural effusion formation, as suggested from the significant decrease in fibrinogen/CRP ratio in the face of a higher FDPs and similar D-dimer concentrations in dogs with pleural effusions compared to control dogs. These results should support the screening of the systemic coagulative state in all dogs with pleural effusion in order to identify those with PHF. Future studies should assess the risk of bleeding in dogs with PHF and pleural effusion and should assess if these dogs may benefit from treatment with anti-fibrinolytic agents.

## Supporting information

S1 File Output file from the software used for plagiarism analysis. (PDF)

S2 File Excel dataset of the patients’ clinical records included in the present study. (XLSX)
