# Supplementary material for: Hemostatic findings of pleural fluid in dogs and the association between pleural effusions and primary hyperfibrino(geno)lysis: A cohort study of 99 dogs
Source: PLoS One. 2018 Feb 20;13(2):e0192371. doi: 10.1371/journal.pone.0192371 (PMC5819782; doi:10.1371/journal.pone.0192371)
Supplement: S1 File — (PDF) [file pone.0192371.s001.pdf]

Hemostatic findings of 12.01.2018

by Andrea Zoia

Submission date: 12-Jan-2018 12:16PM (UTC+0100)
Submission ID: 902113056
File name: Revised_Manuscript.doc (347K)
Word count: 7326
Character count: 42713

Hemostatic findings of pleural fluid in dogs and the association between pleural effusions and primary hyperfibrino(geno)lysis: a cohort study of 99 dogs

Andrea Zoia[1*], Michele Drigo[2], Christine J. Piek[3], Paolo Simioni[4], Marco Caldin[5]

[1] Department of Internal Medicine, San Marco Veterinary Clinic, Padua, Italy

[2] Department of Medicina Animale, Produzione e Salute, Padua University, Legnaro, Italy

[3] Department of Clinical Sciences of Companion Animals, Faculty of Veterinary Medicine, Utrecht University, Utrecht, The Netherlands

[4] Department of Cardiologic, Thoracic and Vascular Sciences, University of Padua Medical School, Padua, Italy

[5] Department of Clinical Pathology, Laboratorio d'Analisi Veterinarie San Marco, Padua, Italy

*Corresponding author

E-mail: zoia.andrea06@googlemail.com (AZ)

Abstract

The primary objective of this study was to determine if activation of coagulation and fibrinolysis occurs in canine pleural effusions. Thirty-three dogs with pleural effusions of different origin were studied. Pleural effusion fibrinogen concentrations were significantly lower, while pleural fibrin-fibrinogen degradation products (FDPs) and D-dimer concentrations were significantly higher than those in plasma ($P < 0.001$ for all comparisons). These results show that, in canine pleural fluids, there is evidence of coagulation activation and fibrinolysis. The secondary aims of the current study were to determine if primary hyperfibrinolysis ([PHF] i.e., elevated plasma FDPs with a normal D-dimer concentrations), occurs in dogs with pleural effusion, and whether the presence of a concurrent inflammatory process may have activated the hemostatic cascade, with its intrinsically linked secondary hyperfibrinolysis, masking the concurrent PHF. The previously 33 selected dogs with pleural effusion (group 1) were compared to two control groups of 33 healthy (group 2) and 33 sick dogs without pleural effusion (group 3). Serum fibrinogen, FDPs, D-dimer, C-reactive protein (CRP), fibrinogen/CRP ratio, and frequency of PHF were determined. Fibrinogen, FDPs, D-dimer and CRP concentrations in group 1 were significantly increased compared to group 2 ($P < 0.001$ for all comparisons). FDPs and CRP concentrations in group 1 were also significantly increased compared to group 3 ($P = 0.001$ and $P < 0.001$, respectively). The fibrinogen/CRP ratio was significantly decreased in group 1 compared to groups 2 and 3 ($P < 0.001$ for both comparison). The frequency of PHF was significantly higher in group 1 compared to groups 2 ($P = 0.004$), but not compared to group 3. These results support the hypothesis that PHF occurs significantly more often in dogs with pleural effusion compared to healthy dogs. Nevertheless, the decrease in the fibrinogen/CRP ratio in group 1 compared to group 3, considering the higher FDPs and similar D-dimer

concentrations, would suggest that PHF is also more frequent in dogs with pleural effusion compared to sick control dogs, and that this phenomenon is hidden due to concurrent secondary hyperfibrinolysis.

Introduction

Fibrinolysis is the process whereby stable fibrin strands are broken down by plasmin [1]. Localized fibrinolysis in response to thrombosis is necessary for the re-establishment of blood flow, and has been termed physiologic fibrinolysis [2]. Pathologic hyperfibrinolysis occurs in disease syndromes that induce increased concentrations of plasminogen activators, decreased concentrations of plasminogen inhibitors, or a combination of both [3]. Primary hyperfibrinolysis (PHF), also sometimes named primary hyperfibrinogenolysis [2,4,5], or pathologic fibrinolysis, [2] develops independently of intravascular activation of coagulation, and plasmin is formed without concomitant formation of thrombin [6]. In PHF, the production of plasmin within the general circulation overwhelms the neutralizing capacity of the antiplasmins, causing generalized fibrinogenolysis, increased production of fibrin-fibrinogen degradation products (FDPs), degradation of coagulation factors V, VIII, IX, XI [4], and degradation of any pre-existing fibrin localized in thrombi and hemostatic clots [4,6,7], potentially leading to severe bleeding [2]. Besides an excessive serum plasmin concentration, other enzymes such as serum tryptase or non-plasmic polymorphonuclear elastase have been reported as possible causes of PHF, when their serum concentration overwhelms the neutralizing capacity of the antiplasmins [8-10]. In humans, this has been associated with acute conditions, such as surgical procedures [11], shock [4], liver transplantation [12], acute leukaemia [13], or treatment with thrombolytic drugs. It can also be observed in chronic conditions such as tumours [14], chronic liver disease [15], or following peritoneovenous shunting [16-19]. Secondary or "reactive" hyperfibrinolysis, on the other hand, is a consequence of activation of coagulation causing generation of thrombin which stimulates the endothelium to produce an increased amount of tissue plasminogen activator [6]. Secondary fibrinolysis is present in virtually every patient with disseminated intravascular coagulation

(DIC), as it is an appropriate response to persistent thrombin generation [2]. Because "cross-talk" between the inflammatory and hemostatic systems may be responsible for the activation of hemostasis associated with systemic inflammation [20,21], secondary or "reactive" hyperfibrinolysis, is often present in patients with inflammatory diseases.

Results of coagulation tests, more specifically FDPs and D-dimer, may help differentiate between either primary or secondary hyperfibrinolysis. During PHF, the production of FDPs is increased but the production of D-dimer is not [4]. Therefore, having elevated plasma FDPs with a normal D-dimer concentration has been suggested in human medicine as a possible indicator of PHF in the clinical setting [4,8,9]. Recently, using this criterion, we have demonstrated that dogs with ascites, above all when resulting from right-sided congestive heart failure, have abnormalities in their coagulation tests, suggesting PHF [22,23].

Pleural effusion is the pathological accumulation of fluid in the pleural space, which is classified following its pathophysiology of formation as exudate, transudate, chylous, and hemorrhagic pleural effusions [24]. Exudates form secondary to increased permeability of the pleural surface affected by inflammatory processes or neoplasia, while transudates are the result of systemic disorders altering Starling forces and form across a normal pleural surface [24,25]. Chylous effusions usually occur due to thoracic duct leak of lymph rich in triglycerides. This lymph forms from the reabsorption of the interstitial fluid of the lower extremities, abdomen (including intestinal lacteals), and thorax and is generally similar to plasma. Hemorrhagic pleural effusions occur secondary to the accumulation of blood in the pleural cavity. Consequently, all pleural effusions contain plasma or an exudate or an ultrafiltrate of plasma. Therefore, pleural effusions may contain all of the proteins/enzymes that are present in plasma, including those that participate in coagulation and fibrinolysis, as already demonstrated for ascitic fluids [26]. Fluids

of virtual cavities, of which a pleural effusion is a pathological manifestation, need to be without clots to allow smooth sliding of organs over each other, and therefore clots need to be rapidly lysed. The above statement is supported by the clinical observation that pleural effusions rarely form clots *in vivo* as shown in an experiment in dogs in which the inoculation of blood or a solution containing fibrinogen and thrombin into the pleural cavity caused the activation of the coagulation system, followed by fibrinolysis [27].

Therefore, the primary objective of the study reported here was to determine if coagulation and fibrinogenolytic/fibrinolytic activity (i.e., low fibrinogen and elevated FDPs and/or D-dimer) occurs in any type of canine pleural effusion. The secondary objectives of this study were to determine if systemic clotting abnormalities suggesting PHF (i.e., elevated plasma FDPs with a normal D-dimer concentration) occurs in these dogs, and whether an inflammatory process present in these dogs may have activated the hemostatic cascade, along with its intrinsically linked secondary hyperfibrinolysis, possibly masking concurrent PHF.

Materials and Methods

Animals

In this cohort study, the fibrinogenolytic/fibrinolytic activity of pleural effusions and the frequency of PHF in dogs with pleural effusion was compared to control dogs without pleural effusion. Group 1 included dogs with any type of pleural effusion, confirmed by thoracic radiography, ultrasonography or computer tomography, which consecutively presented to the San Marco Veterinary Clinic from September 2011 to February 2014. Dogs were only included in the study if the pleural effusion was collected and analyzed, and the pathophysiologic cause of the pleural effusion was determined. Two control groups were created. Group 2 included

clinically-healthy dogs coming to the clinic for routine annual check-ups, elective surgery, blood donor health screening programs, and pre-breeding examinations, and group 3 included sick dogs without pleural effusion. Dogs in both groups 2 and 3 were chosen with the use of the electronic medical database P.O.A System-Plus 9.0® from all dogs that presented to the San Marco Veterinary Clinic in the period between May 2004 and February 2014 using the following procedure. All control dogs were individually matched to group 1 dogs for age (± 6 months), sex (including neuter status), and breed. When a breed match of the same age and sex of a dog in group 1 was not found in the database, a dog with similar weight (± 5 kg) was included instead. The control dogs from groups 2 and 3 were selected as closely as possible to the admission date of the corresponding group 1 dog to reduce drift in the laboratory results. When two or more dogs fulfilled these criteria, the choice was randomly made by the computer system P.O.A System-Plus 9.0®. Dogs of all three groups were included in the study only if there was a complete medical record, including history and results of physical examination, complete blood count (including blood smear examination), serum biochemistry analysis, coagulation profile analysis, and urinalysis. For groups 1 and 3, the determination of a specific diagnosis for the presenting complaint was also required for inclusion in the study. Dogs of all three groups were excluded from the study if they presented with a concomitant abdominal effusion or if they had been treated with plasma, plasma derivate or anticoagulant therapy/intoxication within 30 days before study enrolment.

Pleural effusion classification in group 1 dogs

Pleural effusions in group 1 dogs were classified according to their pathophysiology of formation. In accordance with the Starling's law [28], transudates result from decreased colloid

osmotic pressure (COP) (i.e., dogs with severe hypoproteinemia) or increased hydrostatic pressure (i.e., dogs with diseases causing venous hypertension), and exudates result from increased vascular permeability (i.e., dogs with inflammatory or neoplastic diseases directly involving the thoracic wall and/or serosal surfaces). Since COP poorly correlates with albumin in sick human patients [29], while correlating well with serum total protein in healthy dogs [30,31], hypoproteinemia was used to help identify diseases causing a decreased COP. Pleural effusion caused by blood vessel rupture was classified as a hemorrhagic effusion (i.e., dogs with iatrogenic, traumatic or spontaneous intra-thoracic bleeding). Finally, chylous effusions resulted from disruption of the thoracic duct or its tributaries [32]. Fluid triglycerides >110 mg/dL and fluid to serum triglyceride ratio > 1 were also used to identify diseases causing chylous effusion [33,34]. Diagnosis of the disease causing the pleural effusion was used as the criterion standard to establish the pathophysiology of the pleural fluid formation.

Pleural effusion collection and measurement of parameters

A 10 mL sample of pleural effusion was collected at the time of presentation from each group 1 dog via ultrasonographic-guided thoracentesis. Fluid samples were transferred in plastic tubes with K_3-EDTA for the determination of haematocrit and total nucleated cell count, and in plain glass tubes for the determination of total protein with an automated chemistry analyzer (Olympus AU 2700, Olympus Diagnostics, Hamburg, Germany). A board certified clinical pathologist performed the cytology examinations on all fluid samples. Fluid samples were also transferred in a 3.5 mL plastic tubes with 3.2% sodium citrate (final ratio of volume of anticoagulant to volume of blood, 1:9) (3.2% sodium citrate Vacuette® 3.5 mL, Grenier Bio-One, Kremsmünster, Austria) for the measurement of coagulation variables (see next paragraph).

Plasma and pleural effusion coagulation parameter analysis

Coagulation profile analysis was performed for all dogs and included the determination of fibrinogen, FDPs, and D-dimer. A venous blood sample was taken from the cephalic (for medium/large-size dogs) or jugular (for small size-dogs) veins for routine laboratory analysis. In dogs of group 1, venous blood samples were taken at the same time (± 2 hours) as the pleural effusion samples were collected. A 3.5 mL aliquot of this blood was transferred in a plastic tube with 3.2% sodium citrate with a final ratio of volume of anticoagulant to volume of blood of 1:9 (3.2% sodium citrate Vacuette® 3.5 mL, Grenier Bio-One, Kremsmünster, Austria) for the measurement of coagulation parameters. Tubes with sodium citrate were centrifuged at 1,950 *g* for 5 min, plasma was harvested, and coagulation profile analysis was performed within 1 hour after blood sample collection. Pleural effusion and plasma fibrinogen concentrations were determined via quantitative assays (STA Fibrinogen, Diagnostica Stago, Asnières sur Seine, France), with an automated analyzer (STA-R Evolution, Diagnostica Stago, Roche, Bäsel, Switzerland). The detection limit for fibrinogen concentration was 60 mg/dL; for statistical analysis values below this concentration were entered into the data sheet as 59 mg/dL. Pleural effusion and plasma concentrations of FDPs were measured with an immunoturbidimetric quantitative assay (STA - Liatest® FDP, Diagnostica Stago, Asnières sur Seine, France), with an automated analyzer (STA-R Evolution, Diagnostica Stago, Roche, Bäsel, Switzerland). Pleural effusion and plasma D-dimer concentrations were determined via a validated [35], immunoturbidimetric quantitative assay (Tina-quant D-Dimer, Roche Diagnostic GmbH, Mannheim, Germany), with an automated analyzer (Olympus AU 2700, Olympus Diagnostics, Hamburg, Germany).

All collection procedures were performed for the dog's benefit and for standard diagnostic and monitoring purposes. Previous informed written consent was obtained from all dog owners. Anaesthesia, euthanasia, or any kind of animal sacrifice were not required for any part of the study.

Primary hyperfibrinogenolysis

Primary hyperfibrinogenolysis was defined as a discordant result between changes in plasma FDPs and D-dimer concentrations, with FDPs above the reference interval (reference interval, < 5 μg/mL) and D-dimer within the reference interval (reference interval 0.01 to 0.34 μg/mL).

C-reactive protein

The serum C-reactive protein (CRP) concentration was measured with an automated analyzer (Olympus AU 2700, Olympus Diagnostics, Hamburg, Germany), using an immunoturbidimetric assay validated in humans (CRP OSR6147, Olympus Life and Material Science Europe GmbH, Lismeehan, O'Callaghan's Mills, Ireland), that showed a good correlation (r = 0.98) [36] with those of a previously validated canine-specific ELISA (Tridelta Phase range canine CRP kit, Tridelta Development Ltd., Brey, Ireland).

Statistical analysis

Kolmogorov-Smirnov test was used to assess if data were normally distributed. In group 1 only (dogs with pleural effusion), fibrinogen, FDPs, and D-dimer concentrations were compared between pleural effusion and venous blood via the Wilcoxon signed ranks test. Serum fibrinogen, plasma FDPs, plasma D-dimer, serum CRP concentrations, and the serum fibrinogen/CRP ratio were then compared between group 1, group 2 (healthy dogs), and group 3 (sick dogs without pleural effusion) via ANOVA (fibrinogen concentrations) followed by Tamhane post-hoc test or via Kruskal-Wallis tests (D-dimer, FDPs, CRP concentrations, and fibrinogen/CRP ratio) followed by a Mann-Whitney test. The Fisher's exact test was used to assess differences among and between the three groups in frequency of PHF as previously defined (i.e., elevated plasma concentration of FDPs along with a normal plasma D-dimer concentration).

For all statistical analyses, values of $P < 0.05$ were considered significant.

Results

Animals

During the study period, 156 dogs with any type of pleural effusion, as confirmed by thoracic radiography, ultrasonography or computer tomography, presented to the clinic. In 59 of these dogs, pleural effusion and venous blood samples were collected at presentation. Twenty-six dogs (nine due to rodenticide exposure, 14 with concurrent abdominal effusion, and three due to lack of identification of the underlying cause for the pleural effusion formation) were excluded from further analysis. The remaining 33 dogs entered the study in group 1. Eighteen dogs were male (15 sexually intact and three neutered) and 15 female (11 sexually intact and four spayed). There were five mongrel dogs, three German shepherd dogs and three Labrador retrievers. Each

of the following breeds was represented by two dogs: boxer, Deutsch kurzhaar, greyhound, pug, Rottweiler, and West Highland white terrier. The remaining nine dogs were of other breeds. One dog had pleural effusion due to decreased COP, four due to increased hydrostatic pressure, 20 due to increased vascular permeability, four due to chylous effusion, and four due to haemorrhage. The underlying diseases leading to pleural effusion formation are summarized in Table 1.

Group 2 (clinically healthy dogs) was 100% matched for sex and neutering status to group 1. Group 2 was 76% breed matched to group 1; the breed match was incomplete in 8 out of 33 dogs.

Group 3 (sick dogs without pleural effusion) was 100% matched for sex and neutered status to group 1. Group 3 was 91% breed matched to group 1; breed match was incomplete in 3 out of 33 dogs. Causes of sickness included gastrointestinal disorders (n = 6), brain diseases (5), endocrinopathies (4), sepsis and infectious diseases (5), neoplastic diseases (2), compression of the spinal cord by intervertebral disks (2), protein losing nephropathy (2), and other causes (6).

There was no statistical difference regarding age between groups 1 (7.36 ± 4.13 years; range, 0.33-16.42 years), group 2 (7.05 ± 4.10 years; range, 0.33-14.75 years) and group 3 (7.30 ± 4.06 years; range, 0.33-16.42 years; F = 0.053; P = 0.95).

Table 1. Causes of pleural effusions formation in 33 dogs with pleural effusion

**Group 1 Dogs with pleural effusion (n = 33)**				
**↓ COP transudates (n = 1)**	**↑ HP transudates (n = 4)**	**Exudates (n = 20)**	**Chylous PE (n = 4)**	**Hemorrhagic PE (n = 4)**
PLE (1)	Neoplasia non directly involving the pleural surfaces	Pyothorax (11)<br>Neoplasia directly involving the	Idiopathic (3)<br>Cardiogenic (1)	Malignancies (4)

	(3)<br>Cardiogenic (1)	pleural surfaces (8)<br>Non septic PE (1)		

↓ COP, decreased colloid osmotic pressure; ↑ HP, increased hydrostatic pressure; PLE, protein losing enteropathy; PE, pleural effusion

Pleural effusion and plasma coagulation parameters analysis in dogs of group 1

Pleural effusion fibrinogen concentrations (median = 59 mg/dL; range: 59-59) were significantly lower ($P < 0.001$) than those in the plasma (median = 419 mg/dL; range: 131-1406; reference interval: 152-284 mg/dL; Fig 1), and they were below the instrument detection limit (i.e., < 60 mg/dL) in all 33 dogs. When the pleural effusion fibrinogen concentration of each dog was compared to its matching plasma value, the pleural effusion fibrinogen concentration was lower than the plasma sample in all dogs.

The quantitative FDP concentrations in the pleural effusion (median = 151 mg/dL; range: 0.69-151) were significantly ($P < 0.001$) higher than plasma quantitative FDP concentrations (median = 5.55 mg/dL; range: 0.83-108.47; Fig 2), and they were higher than the plasma reference value (< 5 µg/mL) in all 33 pleural effusions. When the pleural effusion of quantitative FDPs concentration of each dog was compared to its matching plasma value, the pleural effusion FDPs concentration was lower than the plasma sample in all dogs.

Pleural effusion D-dimer concentrations were significantly ($P < 0.001$) higher (median: 3.84 µg/mL; range: 0.05-9.61) than they were in the plasma (median: 0.07 µg/mL; range: 0.01-7.69; Fig 3), and they were higher than the plasma reference interval (0.01-0.34 µg/mL) in 28 out of the 33 pleural effusions. When the pleural effusion D-dimer concentration of each dog was

compared to its matching plasma value, the pleural effusion D-dimer concentration was higher than the plasma sample concentration in 32 cases and lower in one case (i.e., in a hemorrhagic pleural effusion).

In conclusion, all the pleural effusions had non-measurable fibrinogen concentrations and all the 33 pleural effusions had at least one fibrin/fibrinogenolytic marker above the reference interval of what is normal for a plasma sample.

Fig 1. Tukey boxplots of plasma and pleural effusion fibrinogen concentrations of the 33 dogs of group 1. [a,b]Data distributions with different letters are significantly different ($P < 0.001$). The bottom and top of the box are the 1st and 3rd quartiles; the median is the band inside the box. Whiskers correspond to the lowest datum still within 1.5 interquartile range of the lower quartile, and the highest datum still within 1.5 interquartile range of the upper quartile. Circles and stars are outlier and extreme outlier values (more than 1.5 and 3 interquartile range away from the closest end of the box, respectively).

Fig 2. Tukey boxplots of plasma and pleural effusion quantitative FDPs concentrations of the 33 dogs of group 1. [a,b]Data distributions with different letters are significantly different ($P < 0.001$). See Fig 1 for remainder of key.

FDPs, Fibrin-fibrinogen degradation products.

Fig 3. Tukey boxplots of plasma and pleural effusion D-dimer concentrations of the 33 dogs of group 1. [a,b]Data distributions with different letters are significantly different ($P < 0.001$). See Fig 1 for remainder of key.

Fibrinogen, FDPs, D-dimer, frequency of PHF, CRP, and fibrinogen/CRP ratio

Plasma fibrinogen concentrations (reference interval, 152 to 284 mg/dL) were significantly ($P < 0.001$) higher in group 1 (mean ± SD, 469.21 ± 289.88 mg/dL; 95% CI, 366.43 to 572.00 mg/dL) versus group 2 (mean ± SD, 223.52 ± 58.55 mg/dL; 95% CI, 202.75 to 244.28 mg/dL), while they were not significantly different ($P = 0.559$) between group 1 and group 3 (mean ± SD, 395.79 ± 204.21 mg/dL; 95% CI, 323.28 to 468.20 mg/dL). Plasma fibrinogen concentrations were also significantly ($P < 0.001$) lower for group 2 versus group 3; (Fig 4).

Plasma concentrations of FDPs (reference interval, < 5 μg/mL) were significantly higher ($P < 0.001$) for group 1 (median, 5.55 μg/mL; range, 0.83 to 108.47 μg/mL) versus groups 2 (median, 0.97 μg/mL; range, 0.10 to 6.61 μg/mL) and they were also significantly higher ($P = 0.001$) versus group 3 (median, 2.24 μg/mL; range, 0.10 to 28.21 μg/mL). Plasma concentrations of FDPs were also significantly ($P = 0.001$) lower for group 2 versus group 3; (Fig 5).

Plasma D-dimer concentrations (reference interval 0.01 to 0.34 μg/mL) were significantly higher ($P < 0.001$) for group 1 (median, 0.07 μg/mL; range, 0.01 to 7.69 μg/mL) versus group 2 (median, 0.04 μg/mL; range, 0.01 to 0.49 μg/mL; $P < 0.001$), while they were not significantly different ($P = 0.964$) between group 1 and group 3 (median, 0.10 μg/mL; range, 0.01 to 0.88 μg/mL). Plasma D-dimer concentrations were also significantly ($P < 0.001$) lower for group 2 versus group 3; (Fig 6).

Primary hyperfibrinogenolysis (i.e., elevated plasma concentration of FDPs with a normal plasma D-dimer concentration) was detected for 13 (39.4%) dogs in group 1, two (6.1%)

dogs in group 2, and eight (24.2%) dogs in group 3. Frequency of PHF was significantly (P = 0.002) higher for group 1 versus groups 2. There were no significant differences in frequency of PHF between group 1 and group 3 (P = 0.29) and between group 2 and group 3 (P = 0.08). Of the 13 dogs in group 1 with PHF, one had pleural effusion due to increased hydrostatic pressure, nine due to increased vascular permeability, two due to chylous effusion, and two due to haemorrhage. The only dog included in the study with a transudative pleural effusion due to decreased COP did not have PHF. Finally, of the 13 dogs in group 1 with PHF, one had concurrent hypofibrinogenemia. None of the 11 dogs in groups 2 or 3 with PHF had hypofibrinogenemia.

Serum CRP concentration (reference interval, 0.01 to 0.22 mg/dL) was significantly increased in group 1 (median, 5.31 mg/dL; range, 0.01 to 14.57 mg/mL) compared to group 2 (median, 0.02 mg/dL; range, 0.01 to 0.08 mg/mL) and 3 (median, 0.51 mg/dL; range, 0.01 to 10.78 mg/mL) ($P < 0.001$ for both comparison). Serum CRP concentration was also significantly lower for group 2 versus group 3 ($P < 0.001$); (Fig 7).

Serum fibrinogen/CRP ratio was significantly decreased in group 1 (median, 82 mg/dL; range, 21 to 13600 mg/mL) compared to group 2 (median, 12250 mg/dL; range, 281 to 28400 mg/mL) and 3 (median, 678 mg/dL; range, 50 to 41300 mg/mL) ($P < 0.001$ for both comparison). Serum fibrinogen/CRP ratio was also significantly lower for group 2 versus group 3 ($P < 0.001$); (Fig 8).

Fig 4. Tukey boxplots of plasma fibrinogen concentrations from dogs with pleural effusion (n = 33), healthy dogs (33), and sick dogs without pleural effusion (33). [a,b]Data distributions

with different letters are significantly different ($P < 0.001$ for both comparisons). See Fig 1 for remainder of key.

a b c

Fig 5. Tukey boxplots of plasma FDP concentrations from dogs with pleural effusion (n = 33), healthy dogs (33), and sick dogs without pleural effusion (33). [a,b,c]Data distributions with different letters are significantly different ($P < 0.001$ for "a,b" comparison; $P = 0.001$ for "a,c" and "b,c" comparisons). See Fig 1 for remainder of key.

Fig 6. Tukey boxplots of plasma D-dimer concentrations from dogs with pleural effusion (n = 33), healthy dogs (33), and sick dogs without ascites (33). [a,b]Data distributions with different letters are significantly different ($P < 0.001$ for both comparisons). In the graph, in group 1, it is missing an extreme outlier with a value of 7.69. See Fig 1 for remainder of key.

Fig 7. Tukey boxplots of plasma CRP concentrations from dogs with pleural effusion (n = 33), healthy dogs (33), and sick dogs without ascites (33). [a,b,c]Data distributions with different letters are significantly different ($P < 0.001$ for all comparisons). See Fig 1 for remainder of key. CRP, C-reactive protein.

Fig 8. Tukey boxplots of plasma fibrinogen/CRP ratio from dogs with pleural effusion (n = 33), healthy dogs (33), and sick dogs without ascites (33). [a,b,c]Data distributions with different letters are significantly different ($P < 0.001$ for all comparisons). See Fig 1 for remainder of key. CRP, C-reactive protein.

Discussion

The primary aim of this cohort study was to investigate whether coagulation and fibrinogenolytic/fibrinolytic activity occurs in any type of canine pleural effusion. The results of the current study show that a) in the pleural effusion of dogs, there is evidence of coagulation activation and fibrinolysis in every case, and b) this phenomenon occurs independently of the underlying mechanism that leads to pleural effusion formation.

The first statement is supported by the finding that fibrinogen concentrations in dogs are undetectable and significantly lower in the pleural effusion, in comparison to plasma, while FDPs and D-dimer pleural fluid concentrations are significantly higher than those in the plasma. Taken together, these results would suggest that fibrinogen, upon entrance into the pleural cavity, is converted into cross-linked fibrin and then lysed to form FDPs and D-dimer, or that some fibrinogen, fibrin monomer or polymers may get lysed even before being transformed in cross-linked fibrin in FDPs. This latter hypothesis would explain the few pleural effusions with increased FDPs but with D-dimer concentrations lower than the plasma reference interval or lower compared to their corresponding plasma value. Similar conclusions have been reached by experiments in a canine model [27,37] and in the human clinical setting [38-40].

To assess if the fibrinogenolytic/fibrinolytic activity of the pleural fluid is independent of the type of pleural effusion, we included in this study effusions formed secondary to five different pathophysiological mechanisms of fluid formation (i.e., transudates due to increased hydrostatic pressure, transudates due to decreased COP, exudates, chylous and hemorrhagic pleural effusions). In the 33 included cases, the pleural fluid fibrinogen concentrations were lower than their corresponding plasma concentrations, and in all cases at least one of the fibrin/fibrinogenolytic markers in the pleural effusions were above the reference interval of what

is considered normal in a plasma sample and higher than their corresponding plasma values. These findings confirm that the fibrinogenolytic/fibrinolytic activity of pleural effusion is independent of the underlying mechanism of its formation. Therefore, pleural effusions have inherent fibrin/fibrinogenolytic activity, as has been shown in the case for human [26,41,42], and ascitic equine fluid [43]. In addition, in a recent study on dogs with ascites formed secondary to different pathophysiological mechanisms, we found that also canine abdominal effusions have the same inherent fibrin/fibrinogenolytic activity [44]. The hypothesis that pleural fluid is inherently fibrinolytic is also supported not only by the clinical observation that pleural effusions rarely form clots *in vivo*, but also by several studies. The first study on the topic was conducted in 1916 and showed that the inoculation of blood or of a solution containing fibrinogen and thrombin into the pleural cavity of dogs caused the activation of the coagulation system followed by rapid fibrinogenolysis/fibrinolysis, allowing the inoculated blood to remain fluid to a large extent [27]. These initial findings were later confirmed by another experimental study in dogs [37], and by studies in humans with hemothorax [45,46], and by other studies including patients with different types of pleural effusion [47,48]. The activation of plasminogen in the pleural effusion, responsible for the documented fibrinogenolysis/fibrinolysis, may be caused by the presence of tissue plasminogen activator, urinary plasminogen activator, both enzymes, or other fibrinolytic enzymes [46,48], which can be released (from a preformed storage pool) or leak into the pleural fluid (following the damage induced by the disease causing the pleural effusion) from the mesothelial and submesothelial endothelium, inflammatory or neoplastic cells.

The secondary aim of this cohort study was to determine if systemic clotting abnormalities suggesting PHF (i.e., elevated plasma FDPs with a normal D-dimer concentration) occur in dogs with pleural effusion, and whether a possible inflammatory process present in

these dogs may have activated the hemostatic cascade, along with its intrinsically linked secondary hyperfibrinolysis, possibly masking this phenomenon. The results show that a) PHF, according to our definition, occurs significantly more often in dogs with pleural effusion compared to healthy dogs, and b) although there was also a trend for increased PHF in dogs with pleural effusion compared to sick dogs, this difference did not reach significance. Nevertheless, the plasma fibrinogen concentration in dogs with pleural effusion was similar to sick control dogs (Fig 4), despite a significantly increased serum CRP concentration in comparison to these dogs (Fig 6). The resulting significant decrease in the fibrinogen/CRP ratio in dogs with pleural effusion compared to the sick control group, in the face of higher FDPs concentration and a similar D-dimer concentration, would suggest that PHF is also more frequent in dogs with pleural effusion compared to sick control dogs. Therefore, similarly to dogs with ascites [23], dogs with pleural effusion are at increased risk of PHF, which is the cause of the relative (in comparison to their inflammatory state) decreased fibrinogen concentration observed in these dogs.

In a recent study on dogs with ascites, we found that PHF occurred with different frequency in all dogs with ascites. Moreover, we found that PHF was statistically more frequent in dogs with ascites secondary to an increase in hydrostatic pressure compared to dogs in which the ascites formed secondary to another pathophysiological method. In addition, dogs with ascites secondary to an increase in hydrostatic pressure were the only dogs to have a statistically more frequent PHF in comparison to both healthy and sick dogs without ascites [23]. The results of this study showed that PHF also occurred with different frequency in all dogs with pleural effusion, with the exception of the dogs in which pleural effusion formed secondary to decreased colloid osmotic pressure. Due to the small number of dogs with pleural effusion included in this

study, statistical analysis to assess if different types of pleural effusion were associated more frequently with PHF was not carried out. Furthermore, our search in the human medical literature found no studies evaluating this issue.

It is interesting to note that two (6.1%) of the healthy dogs had findings consistent with a diagnosis of PHF, but all of them only had mild elevations in FDPs, with normal plasma fibrinogen concentrations. Similar findings have been found in a recent study in a different healthy canine population [23]. Therefore, it is possible that the definition we adopted for PHF is not 100% specific for diagnosing PHF and that more stringent criteria might be required. However, it is also possible that some of our healthy animals had occult/subclinical disease resulting in PHF or that low grade PHF could be a physiological feature detectable with our definition of PHF.

In the normal state, the pleural space is a dynamic compartment in which fluids, electrolytes and proteins are continuously exchanged, with the net direction of this flux being from the parietal pleural capillaries, through the pleural space and into the visceral pleural capillaries and lymphatics. In the pathological state, pleural fluid accumulates within the thoracic cavity when more fluid enters the pleural space than is removed [49,50]. Therefore, pleural fluid, which has traditionally been regarded by physicians as an inert fluid, contains all of the proteins/enzymes that are present in plasma, including those that participate in coagulation and fibrinolysis [39]. Plasma coagulation-relevant proteins in pleural fluids are no longer contained in their natural environment (i.e. blood vessels), but are in a relatively acellular environment (i.e. the pleural space). They are also not exposed to the vascular endothelium where, according to the cell-based model of hemostasis, in which tissue factor-bearing cells and platelets are necessary for the three steps (initiation, amplification and propagation) of hemostasis [51], their actions

would no longer be well-regulated. This lack of regulation could result in the formation of a fluid with an inherently increased fibrinogen/fibrinolytic activity, as already demonstrated to be the case for ascitic fluid [23,26,41,42]. The increased frequency of PHF documented in this study in dogs with pleural effusion, compared to healthy dogs or sick dogs without pleural effusion, might possibly be explained by the fibrinolytic activity of all types of pleural effusion, which upon re-entering the systemic circulation via the thoracic duct and the pulmonary veins might possibly contribute to the systemic hyperfibrinolytic state found in 39.4% of our dogs with pleural effusion.

One limitation of the current study is that the pleural fibrinogen, FDP, and D-dimer concentrations were measured using kits that had been validated only in canine plasma samples and not in canine pleural effusion samples. Nevertheless, several studies in humans and horses also used plasma validated kits for fibrinogen, FDPs, and D-dimer for the determination of these analytes in intracavitary effusions, without apparent problems [41-43]. A second limitation of the current study is that PHF was diagnosed indirectly, by measuring FDPs and D-dimer [4,8,9], while fibrinolytic enzymes responsible for PHF such as plasmin, serum tryptase, or non-plasmic polymorphonuclear elastase were not measured [2,8-10], neither in the pleural effusion nor in the plasma. Therefore, it may be argued that the increased FDP plasma concentrations are the result of their reabsorption from the pleural fluid rather than de novo formation in the plasma secondary to a systemic PHF. However, despite the possibility that some the plasma FDPs in dogs with pleural effusion originate from the pleural effusion, this does not explain the discrepancy found between their plasma FDPs and plasma D-dimer. In fact, if plasma FDP and D-dimer concentrations were derived only from the pleural fluid, we would expect both of them to be increased in a similar proportion since, as demonstrated in this study, both pleural effusion

FDPs and D-dimer concentrations were almost always higher than the plasma reference interval. Conversely, plasma FDPs were increased in 18 of these dogs, while plasma D-dimer was increased in only five cases (data not shown).

Conclusions

In summary, we have shown that pleural effusions have fibrinolytic activity independent of the underlying mechanism of intra-thoracic fluid accumulation, and we also demonstrated that almost 40% of these dogs have systemic coagulative alterations suggesting PHF. The true frequency of PHF in dogs with pleural effusion could have been underestimated in this study due to the concurrent presence of secondary hyperfibrinolysis caused by the inflammatory disease underlying the cause of pleural effusion formation, as suggested from the significant decrease in fibrinogen/CRP ratio in the face of a higher FDPs and similar D-dimer concentrations in dogs with pleural effusions compared to control dogs. These results should support the screening of the systemic coagulative state in all dogs with pleural effusion in order to identify those with PHF. Future studies should assess the risk of bleeding in dogs with PHF and pleural effusion and should assess if these dogs may benefit from treatment with anti-fibrinolytic agents.

Author Contributions

Conceptualization: AZ MD CJP PS MC.

Formal Analysis: AZ MD MC.

Funding Acquisition: CJP MC.

Investigation: AZ MD CJP PS MC.

Methodology: AZ MD CJP PS MC.

Project Administration: AZ CJP MC.

Resources: AZ MC.

Supervision: MD CJP PS MC.

Writing – original draft: AZ MD CJP PS MC.

Writing – review & editing: AZ MD CJP PS MC.

References

1. Carey M, Cressey DM. Hyperfibrinolysis - is it common? Measurement and treatment including the role of thromboelastography. Clin Risk. 2009; 15: 188–191.
2. Rodgers GM. Acquired coagulation disorders. In: Greer JP, Arber DA, Glader B, List AF, Means Jr. RT, Paraskevas F RG, editors. Wintrobe's Clinical Hematology. 13th ed. Lippincott Williams and Wilkins, WoltersKluwer Health; 2014. p. 1186–1217.
3. Zorio E, Gilabert-Estellés J, España F, Ramón LA, Cosín R, Estellés A. Fibrinolysis: the key to new pathogenetic mechanisms. Curr Med Chem. 2008; 15: 923–929.
4. Mazzi G, Raineri A, Lacava E, De Roia D, Santarossa L, Orazi BM. Primary hyperfibrinogenolysis in a patient with anaphylactic shock. Haematologica. 1994; 79: 283–285.
5. Sallah S, Gagnon GA. Reversion of primary hyperfibrinogenolysis in patients with hormone-refractory prostate cancer using docetaxel. Cancer Invest. 2000; 18: 191–196.
6. Hunt BJ, Segal H. Hyperfibrinolysis. J Clin Pathol. 1996; 49: 958.
7. Duran I, Tannock IF. Disseminated intravascular coagulation as the presenting sign of metastatic prostate cancer. J Gen Intern Med. 2006; 21: C6-C8.
8. Sato N, Takahashi H, Shibata A. Fibrinogen/fibrin degradation products and D-dimer in clinical practice: interpretation of discrepant results. Am J Hematol. 1995; 48: 168–174.
9. Song KS, Kim YA, Kim HK, Park Q. Incidence and possible reasons for discordant results between positive FDP and negative D-dimer latex assays in clinical specimens. Yonsei Med J. 1999; 40: 107–111.
10. Lombardini C, Helia RE, Boehlen F, Merlani P. "Heparinization" and hyperfibrinogenolysis by wasp sting. Am J Emerg Med. 2009; 27: 1176.e1-3.

11. von Sommoggy S, Fraunhofer S, Wahba A, Blümel G, Maurer PC. Coagulation in aortofemoral bifurcation bypass grafting. Eur J Vasc Surg. 1991; 5: 247–253.

12. Blankensteijn JD, Schalm SW, Terpstra OT. New aspects of heterotopic liver transplantation. Transpl Int. 1992; 5: 43–50.

13. García Frade LJ, Sureda A, Torrado MC, García Avello A. High plasma urokinase-type plasminogen activator levels are present in patients with acute nonlymphoblastic leukemia. Acta Haematol. 1992; 88: 7–10.

14. Okajima K, Kohno I, Tsuruta J, Okabe H, Takatsuki K, Binder BR. Direct evidence for systemic fibrinogenolysis in a patient with metastatic prostatic cancer. Thromb Res. 1992; 66: 717–727.

15. Violi F, Ferro D, Basili S, Quintarelli C, Musca A, Cordova C, Balsano F. Hyperfibrinolysis resulting from clotting activation in patients with different degrees of cirrhosis. The CALC Group. Coagulation Abnormalities in Liver Cirrhosis. Hepatology. 1993; 17: 78–83.

16. Scott-Coombes DM, Whawell SA, Vipond MN, Crnojevic L, Thompson JN. Fibrinolytic-Activity of Ascites Caused by Alcoholic Cirrhosis and Peritoneal Malignancy. Gut. 1993; 34: 1120–1122.

17. Schölmerich J, Zimmermann U, Köttgen E, Volk BA, Hasler C, Wilms H, et al. Proteases and antiproteases related to the coagulation system in plasma and ascites. Prediction of coagulation disorder in ascites retransfusion. J Hepatol. 1988; 6: 359–363.

18. Patrassi GM, Martinelli S, Sturniolo GC, Cappellato MG, Vicariotto M, Girolami A. Fibrinolytic study in plasma and ascitic fluid of cirrhotic patients before and after ascites concentration; reinfusion technique. Eur J Clin Invest. 1985; 15: 161–165.

19. Lévy VG, Opolon P, Pauleau N, Caroli J. Treatment of ascites by reinfusion of concentrated peritoneal fluid–review of 318 procedures in 210 patients. Postgrad Med J. 1975; 51: 564–566.

20. Levi M, Keller TT, van Gorp E, ten Cate H. Infection and inflammation and the coagulation system. Cardiovasc Res. 2003; 60: 26–39.

21. Smith S. Overview of Hemostasis. In: Weiss DJ, Wardrop KJ, editors. Schalm's Veterinary Hematology. 6th ed. Wiley-Blackwell; 2010. p. 635–53.

22. Zoia A, Augusto M, Drigo M, Caldin M. Evaluation of hemostatic and fibrinolytic markers in dogs with ascites attributable to right-sided congestive heart failure. J Am Vet Med Assoc. 2012; 241: 1336–1343.

23. Zoia A, Drigo M, Simioni P, Caldin M, Piek CJ. Association between ascites and primary hyperfibrinolysis: A cohort study in 210 dogs. Vet J. 2017; 223: 12–20.

24. Burgess LJ. Biochemical analysis of pleural, peritoneal and pericardial effusions. Clin Chim Acta. 2004; 343: 61–84.

25. Kopcinovic LM, Culej J. Pleural, peritoneal and pericardial effusions - a biochemical approach. Biochem medica. 2014; 24: 123–137.

26. Henderson JM, Stein SF, Kutner M, Wiles MB, Ansley JD, Rudman D. Analysis of Twenty-three plasma proteins in ascites. The depletion of fibrinogen and plasminogen. Ann Surg. 1980; 192: 738–742.

27. Denny GP, Minot GR. The coagulation of blood in the pleural cavity. Am J Physiol. 1916; 39: 455–458.

28. Rose BD. Edematous states. In: Rose BD, Post TW, editors. Clinical Physiology of Acid-base and Electrolyte Disorders. New York: McGraw-Hill; 2001. p. 478–534.

29. Grootendorst AF, van Wilgenburg MG, de Laat PH, van der Hoven B. Albumin abuse in intensive care medicine. Intensive Care Med. 1988; 14: 554–557.

30. Navar PD, Navar LG. Relationship between colloid osmotic pressure and plasma protein concentration in the dog. Am J Physiol. 1977; 233: H295-298.

31. Gabel JC, Scott RL, Adair TH, Drake RE, Traber DL. Errors in calculated oncotic pressure of dog plasma. Am J Physiol. 1980; 239: H810-812.

32. Maskell NA, Butland RJ. BTS guidelines for the investigation of a unilateral pleural effusion in adults. Thorax. 2003; 58 Suppl 2: ii8-17.

33. Skouras V, Kalomenidis I. Chylothorax: diagnostic approach. Curr Opin Pulm Med. 2010; 16: 387-393.

34. Fossum TW, Jacobs RM, Birchard SJ. Evaluation of cholesterol and triglyceride concentrations in differentiating chylous and nonchylous pleural effusions in dogs and cats. J Am Vet Med Assoc. 1986; 188: 49–51.

35. Caldin M, Furlanello T, Lubas G. Validation of an immunoturbidimetric D-dimer assay in canine citrated plasma. Vet Clin Pathol. 2000; 29: 51–54.

36. Martínez-Subiela S, Cerón JJ. Validación analítica de técnicas comerciales para la determinación de haptoglobina, proteína C reactiva y amiloide A sérico en caninos. Arch Med Vet. 2005; 37: 61–66.

37. Napoli VM, Symbas PJ, Vroon DH, Symbas PN. Autotransfusion from experimental hemothorax: levels of coagulation factors. J Trauma. 1987; 27: 296–300.

38. Glauser FL, Otis PT, Levine RI, Smith WR. In vitro pleural fluid clottability andfibrinogen content. Chest. 1975; 68: 205–208.

39. Glauser FL, Otis PT, Levine RI, Smith WR. Coagulation factors and fibrinogen in pleural

effusions. Respiration. 1976; 33: 396–402.

40. Widström O, Kockum C, Nilsson BS. Fibrinogen, fibrin(ogen) degradation products and fibrinopeptide A in pleural effusions. High turnover of fibrinogen in pleurisy. Scand J Respir Dis. 1978; 59: 210–215.

41. Agarwal S, Joyner KA Jr, Swaim MW. Ascites fluid as a possible origin for hyperfibrinolysis in advanced liver disease. Am J Gastroenterol. 2000; 95: 3218–324.

42. Diab SM, Fathy EM, Soliman HH, Zaghloul SG. Hyperfibrinolysis in Advanced Liver Disease : Does Ascites Have a Role ? Arab J Gastroenterol. 2005; 6: 124–130.

43. Delgado MA, Monreal L, Armengou L, Ríos J, Segura D. Peritoneal D-dimer concentration for assessing peritoneal fibrinolytic activity in horses with colic. J Vet Intern Med. 2009; 23: 882–889.

44. Zoia A, Drigo M, Piek CJ, Simioni P, Caldin M. Hemostatic Findings in Ascitic Fluid: A Cross-Sectional Study in 70 Dogs. J Vet Intern Med. 2017; 31: 43-50.

45. Broadie TA, Glover JL, Bang N, Bendick PJ, Lowe DK, Yaw PB, Kafoure D. Clotting competence of intracavitary blood in trauma victims. Ann Emerg Med. 1981; 10: 127–130.

46. Schved JF, Gris JC, Gilly D, Joubert P, Eledjam JJ, D'Athis F. [Fibrinolytic activity in traumatic hemothorax fluids]. Ann Fr d'anesthèsie rèanimation. 1991; 10: 104–107.

47. Idell S, Girard W, Koenig KB, McLarty J, Fair DS. Abnormalities of pathways of fibrin turnover in the human pleural space. Am Rev Respir Dis. 1991; 144: 187–194.

48. Philip-Joët F, Alessi MC, Philip-Joët C, Aillaud M, Barriere JR, Arnaud A, Juhan-Vague I. Fibrinolytic and inflammatory processes in pleural effusions. Eur Respir J. 1995; 8: 1352–1356.

49. Boggs DS, Kinasewitz GT. Review: pathophysiology of the pleural space. Am J Med Sci. 1995; 309: 53–59.

50. Porcel JM, Light RW. Diagnostic approach to pleural effusion in adults. Am Fam Physician. 2006; 73: 1211–1220.

51. Hoffman M, Monroe DM 3rd. A Cell-based Model of Hemostasis. Thromb Haemost. 2001; 85: 958–965.

Hemostatic findings of 12.01.2018

ORIGINALITY REPORT

38%	%	38%	%
SIMILARITY INDEX	INTERNET SOURCES	PUBLICATIONS	STUDENT PAPERS

PRIMARY SOURCES

1	A. Zoia, M. Drigo, C.J. Piek, P. Simioni, M. Caldin. "Hemostatic Findings in Ascitic Fluid: A Cross-Sectional Study in 70 Dogs", Journal of Veterinary Internal Medicine, 2017<br>Publication	15%
2	"Research Communications of the 24th ECVIM-CA Congress", Journal of Veterinary Internal Medicine, 2015.<br>Publication	5%
3	Silvestrini, P., A. Zoia, M. Planellas, X. Roura, J. Pastor, J. J. Cerón, and M. Caldin. "Iron status and C-reactive protein in canine leishmaniasis", Journal of Small Animal Practice, 2014.<br>Publication	1%
4	Karandashova, S., G. Florova, A. O. Azghani, A. A. Komissarov, K. Koenig, T. A. Tucker, T. C. Allen, K. Stewart, A. Tvinnereim, and S. Idell. "Intrapleural Adenoviral Delivery of Human PAI-1 Exacerbates TCN-Induced Pleural Injury in Rabbits", American Journal of Respiratory Cell and Molecular Biology, 2012.	1%

Publication

---

5 Wada, Yoshihiro, Mitsuhiro Uchiba, Yoshiaki Kawano, Nobuyuki Kai, Wataru Takahashi, Jiro Honda, Ken-ichiro Tanoue, Yoshihiro Maeda, Yoji Murakami, Masatoshi Eto, and Takahisa Imamura. "Severe bleeding tendency caused by a rare complication of excessive fibrinolysis with disseminated intravascular coagulation in a 51-year-old Japanese man with prostate cancer: a case report", Journal of Medical Case Reports, 2012. 1%

Publication

---

6 Singh, Jasveer, Dheeraj Kapoor, and Manpreet Singh. "Autologous transfusion in a patient with life threatening traumatic haemothorax: Case Report.", Bangladesh Journal of Medical Science, 2013. 1%

Publication

---

7 Zoia, Andrea, and Marco Caldin. "Coagulation status in dogs with naturally occurring Angiostrongylus vasorum infection and primary hyperfibrinolysis : Primary hyperfibrinolysis and angiostrongylosis", Journal of Small Animal Practice, 2015. 1%

Publication

---

8 Spadaro, Aldo. "High circulating D-dimers are associated with ascites and hepatocellular <1%

carcinoma in liver cirrhosis", World Journal of Gastroenterology, 2008.
Publication

9	"Large-volume Paracentesis versus Dialytic Ultrafiltration in the Treatment of Cirrhotic Ascites", QJM: An International Journal of Medicine, 1991<br>Publication	<1%
10	F. Glauser. "In vitro pleural fluid clottability and fibrinogen content", Chest, 08/01/1975<br>Publication	<1%
11	Tvarijonaviciute, A.. "Adiponectin and IGF-1 are negative acute phase proteins in a dog model of acute endotoxaemia", Veterinary Immunology and Immunopathology, 20110315<br>Publication	<1%
12	E. E. McGrath. "A systematic approach to the investigation and diagnosis of a unilateral pleural effusion : Diagnosis of unilateral pleural effusion", International Journal of Clinical Practice, 10/09/2009<br>Publication	<1%
13	Hiromitsu Kitayama, Tomohiro Kondo, Junko Sugiyama, Kazutomo Kurimoto, Yasuhiro Nishino, Michiaki Hirayama, Yasushi Tsuji. "Venous thromboembolism in hospitalized patients receiving chemotherapy for	<1%

malignancies at Japanese community hospital: prospective observational study", BMC Cancer, 2017
Publication

---

14 Jonathan F. McAnulty. "Prospective Comparison of Cisterna Chyli Ablation to Pericardectomy for Treatment of Spontaneously Occurring Idiopathic Chylothorax in the Dog : Comparison of Cisterna Chyli Ablation to Pericardectomy", Veterinary Surgery, 09/2011 <1%
Publication

---

15 Cardile, Anthony P, Carlos J Sanchez, Meghan E Samberg, Desiree R Romano, Sharanda K Hardy, Joseph C Wenke, Clinton K Murray, and Kevin S Akers. "Human plasma enhances the expression of Staphylococcal microbial surface components recognizing adhesive matrix molecules promoting biofilm formation and increases antimicrobial tolerance In Vitro", BMC Research Notes, 2014. <1%
Publication

---

16 "Peritoneovenous Shunting for Ascites", New England Journal of Medicine, 08/21/1980 <1%
Publication

---

17 C. Yenilmez, Z. Ozdemir Koroglu, H. Kurt, M. Yanas, E. Colak, I. Degirmenci, H. V. Gunes. "A <1%

study of the possible association of plasminogen activator inhibitor type 1 4G/5G insertion/deletion polymorphism with susceptibility to schizophrenia and in its subtypes", Journal of Clinical Pharmacy and Therapeutics, 2017

Publication

18	Palmieri, Maria, Stefano Berrettini, Francesca Forli, Patrizia Trevisi, Elisabetta Genovese, Anna Maria Chilosi, Edoardo Arslan, and Alessandro Martini. "Evaluating Benefits of Cochlear Implantation in Deaf Children With Additional Disabilities :", Ear & Hearing, 2012.<br>Publication	<1%
19	Madarame, Haruhiko, Miwa Kurano, Kazuya Fukumura, Taira Fukuda, and Toshiaki Nakajima. "Haemostatic and inflammatory responses to blood flow-restricted exercise in patients with ischaemic heart disease: a pilot study", Clinical Physiology and Functional Imaging, 2013.<br>Publication	<1%
20	"Research Communications of the 25th ECVIM-CA Congress", Journal of Veterinary Internal Medicine, 2015.<br>Publication	<1%
21	D. Scott-Coombes. "Human intraperitoneal	<1%

fibrinolytic response to elective surgery", British Journal of Surgery, 03/1995
Publication

22	Widstrom, O.. "Fibrinolytic and coagulation mechanisms in stages of inflammation: A study of BCG-induced pleural exudate in guinea pig", Thrombosis Research, 19830301<br>Publication	<1 %
23	Milena S. Espíndola, Luana S. Soares, Leonardo J. Galvão-Lima, Fabiana A. Zambuzi, Maira C. Cacemiro, Verônica S. Brauer, Fabiani G. Frantz. "HIV infection: focus on the innate immune cells", Immunologic Research, 2016<br>Publication	<1 %
24	G. Koren. "A Systematic Review of Lidocaine-Prilocaine Cream (EMLA) in the Treatment of Acute Pain in Neonates", PEDIATRICS, 02/01/1998<br>Publication	<1 %
25	Klenner, S., N. Bauer, and A. Moritz. "Evaluation of Three Automated Human Immunoturbidimetric Assays for the Detection of C-Reactive Protein in Dogs", Journal of Veterinary Diagnostic Investigation, 2010.<br>Publication	<1 %
26	J-P Yared. "Albumin vs. non-protein colloids for fluid resuscitation: does it matter?", Clinical	<1 %

Intensive Care, 12/1/2000
Publication

27	Idell, S., W. Girard, K. B. Koenig, J. McLarty, and D. S. Fair. "Abnormalities of Pathways of Fibrin Turnover in the Human Pleural Space", American Journal of Respiratory and Critical Care Medicine, 1991.<br>Publication	<1%
28	Page R Painter. Theoretical Biology and Medical Modelling, 2005<br>Publication	<1%
29	B. W. J. Hellebrekers. "Pathogenesis of postoperative adhesion formation", British Journal of Surgery, 11/2011<br>Publication	<1%
30	Management of Bleeding Patients, 2016.<br>Publication	<1%
31	David T. Runk. "Measurement of Plasma Colloid Osmotic Pressure in Normal Thoroughbred Neonatal Foals", Journal of Veterinary Internal Medicine, 09/2000<br>Publication	<1%
32	V. E. O. (Ted) Valli, Matti Kiupel, Dorothee Bienzle, R. Darren Wood. "Hematopoietic System", Elsevier BV, 2016<br>Publication	<1%

33	Adam Auckburally. "A comparison of induction of anaesthesia using a target-controlled infusion device in dogs with propofol or a propofol and alfentanil admixture", Veterinary Anaesthesia and Analgesia, 07/2008<br>Publication	<1%
34	Komissarov, A. A., G. Florova, A. Azghani, S. Karandashova, A. K. Kurdowska, and S. Idell. "Active -macroglobulin is a reservoir for urokinase after fibrinolytic therapy in rabbits with tetracycline-induced pleural injury and in human pleural fluids.", AJP Lung Cellular and Molecular Physiology, 2013.<br>Publication	<1%
35	"Abstracts of free papers presented at the annual meeting of the Obstetric Anaesthetists' Association, Edinburgh, 26 - 27 May 2011", International Journal of Obstetric Anesthesia, 201105<br>Publication	<1%
36	Momin, Takki A., Robyn A. Macsata, and Anton N. Sidawy. "Thrombogenesis and Thrombolysis", Haimovici s Vascular Surgery Ascher/Haimovici s Vascular Surgery, 2012.<br>Publication	<1%
37	Karkhanis, Vinaya, and Jyotsna Joshi. "Pleural effusion: diagnosis, treatment, and	<1%

management", Open Access Emergency Medicine, 2012.
Publication

---

38 D. Feller-Kopman. "Assessment of Pleural Pressure in the Evaluation of Pleural Effusions", Chest, 01/01/2009 <1%
Publication

---

39 Yokokawa, Tetsuro, Yasuo Sugano, Akito Shimouchi, Atsushi Shibata, Naoya Jinno, Toshiyuki Nagai, Hideaki Kanzaki, Takeshi Aiba, Kengo Kusano, Mikiyasu Shirai, Yasuchika Takeishi, Satoshi Yasuda, Hisao Ogawa, and Toshihisa Anzai. "Exhaled Acetone Concentration Is Related to Hemodynamic Severity in Patients With Non-Ischemic Chronic Heart Failure", Circulation Journal, 2016. <1%
Publication

---

40 Tommaso Furlanello,. "Stability of stored canine plasma for hemostasis testing", Veterinary Clinical Pathology, 06/2006 <1%
Publication

---

41 Stephen H. Caldwell. "Coagulation disorders and hemostasis in liver disease: Pathophysiology and critical assessment of current management", Hepatology, 10/2006 <1%
Publication

---

42 CÜCE, Gökhan, SÖZEN, Mehmet Enes,

ÇETİNKAYA, Seda, CANBAZ, Halime Tuba, SEFLEK, Hatice and KALKAN, Serpil. "Effects of Nigella sativa L. seed oil on intima-media thickness and Bax and Caspase 3 expression in diabetic rat aorta", AVES Yayıncılık, 2016.
Publication

<1%

---

43 Meléndez-Lazo, A., A. Tvarijonaviciute, J.J. Cerón, M. Planellas, and J. Pastor. "Evaluation of the Relationship between Selected Reticulocyte Parameters and Inflammation determined by Plasma C-reactive Protein in Dogs", Journal of Comparative Pathology, 2015.
Publication

<1%

---

44 Ch. Pechlaner. "Plasminogen Activators in Inflammation and Sepsis", Acta Medica Austriaca, 7/2002
Publication

<1%

---

45 Marnie L. Campbell. "Burial Duration and Frequency Influences Resilience of Differing Propagule Types in a Subtidal Seagrass, Posidonia australis", PLOS ONE, 2016
Publication

<1%

---

46 Reddy, V.M.. "Hemodynamic Effects of Epinephrine, Bicarbonate and Calcium in the Early Postnatal Period in a Lamb Model of Single-Ventricle Physiology Created In Utero",

<1%

Journal of the American College of Cardiology, 199612
Publication

47 Swati Agarwal. "Ascites fluid as a possible origin for hyperfibrinolysis in advanced liver disease", The American Journal of Gastroenterology, 11/2000 <1%
Publication

48 &NA;. "Current World Literature :", Current Opinion in Pulmonary Medicine, 09/2011 <1%
Publication

49 Zoia, A.. "A new approach to pleural effusion in cats: markers for distinguishing transudates from exudates", Journal of Feline Medicine and Surgery, 200910 <1%
Publication

50 Li, S.. "Gingival bleeding as a presenting sign of primary fibrinogenolysis", Oral Surgery, Oral Medicine, Oral Pathology, Oral Radiology and Endodontology, 201107 <1%
Publication

51 H. KOTHARI. "Plasmin enhances cell surface tissue factor activity in mesothelial and endothelial cells", Journal of Thrombosis and Haemostasis, 01/2009 <1%
Publication

Brian L. Henry. "Recent Research

52 Developments in the Direct Inhibition of Coagulation Proteinases - Inhibitors of the Initiation Phase", Cardiovascular & Hematological Agents in Medicinal Chemistry (Formerly Current Medicinal Chemistry - Cardiovascular & Hematological Agents), 10/01/2008 — <1%
Publication

---

53 V. Toschi, G.M. Rocchini, A. Motta, G.F. Fiorini, C. Cimminiello, F. Violi, C. Castelli, D. Sironi, A. Gibelli. "The hyperfibrinolytic state of liver cirrhosis: possible pathogenetic role of ascites", Biomedicine & Pharmacotherapy, 1993 — <1%
Publication

---

54 Tracy Stokol. "Plasma D-dimer for the diagnosis of thromboembolic disorders in dogs", Veterinary Clinics of North America: Small Animal Practice, 2003 — <1%
Publication

---

55 "Abstracts", Veterinary Dermatology, 11/2008 — <1%
Publication

---

56 Pease, C. T., J. N. Fordham, M. M. Stubbs, and J. R. Archer. "IgA Antibodies to Gram-negative Bacteria in the Serum and Saliva of Patients with Ankylosing Spondylitis", Scandinavian Journal of Rheumatology, 1987. — <1%
Publication

---

57 N A Maskell. "BTS guidelines for the investigation of a unilateral pleural effusion in adults", Thorax, 5/1/2003 <1%

Publication

---

58 "Oral Research Communications of the 23rd ECVIM-CA Congress", Journal of Veterinary Internal Medicine, 2013. <1%

Publication

---

59 Zhang, Xiang-Hui, Mei Zhang, Jia He, Yi-Zhong Yan, Jiao-Long Ma, Kui Wang, Ru-Lin Ma, Heng Guo, La-Ti Mu, Yu-Song Ding, Jing-Yu Zhang, Jia-Ming Liu, Shu-Gang Li, Qiang Niu, Dong-Sheng Rui, and Shu-Xia Guo. "Comparison of Anthropometric and Atherogenic Indices as Screening Tools of Metabolic Syndrome in the Kazakh Adult Population in Xinjiang", International Journal of Environmental Research and Public Health, 2016. <1%

Publication

---

60 Shigeki Kushimoto. "Implications of Fibrinogenolysis in Patients with Closed Head Injury", Journal of Neurotrauma, 04/2003 <1%

Publication

---

61 KORKMAZ, Özlem, GÖKŞEN, Damla, ÖZEN, Samim and DARCAN, Şükran. "Is anti-Mullerian hormone an indicator of potential <1%

polycystic ovary syndrome in prepubertal girls with simple obesity?", Türk Pediatri Derneği, 2016.

Publication

---

62 L SOLANO-GALLEGO. "Molecular Survey of Ehrlichia canis and Anaplasma phagocytophilum from Blood of Dogs in Italy", Annals of the New York Academy of Sciences, 10/01/2006 <1%

Publication

---

63 Furlanello, T.. "Clinicopathological findings in naturally occurring cases of babesiosis caused by large form Babesia from dogs of northeastern Italy", Veterinary Parasitology, 20051125 <1%

Publication

---

64 YILMAZ, Zeki, KOCATÜRK, Meriç, ERALP İNAN, Oya and LEVENT, Pınar. "Thromboelastographic evaluation of hemostatic functionin dogs with dilated cardiomyopathy", TÜBİTAK, 2017. <1%

Publication

---

65 Ann Kristin Barton, Caroline Wirth, Angelika Bondzio, Ralf Einspanier, Heidrun Gehlen. "Are pulmonary hemostasis and fibrinolysis out of balance in equine chronic pneumopathies?", Journal of Veterinary Science, 2017 <1%

Publication

66 Martinez-Subiela, S.. "Acute phase protein response in experimental canine leishmaniosis", Veterinary Parasitology, 20110825 <1%

Publication

67 Planellas, Marta, Rafaela Cuenca, Maria-Dolores Tabar, Coralie Bertolani, Cyrill Poncet, Josep M Closa, Juan Lorente, Jose J Cerón, and Josep Pastor. "Evaluation of C-reactive protein, Haptoglobin and cardiac troponin 1 levels in brachycephalic dogs with upper airway obstructive syndrome", BMC Veterinary Research, 2012. <1%

Publication

68 Alexandre Merlo. "Serum amyloid A is not a marker for relapse of multicentric lymphoma in dogs", Veterinary Clinical Pathology, 3/2008 <1%

Publication

69 "WORLD TRANSPLANT CONGRESS 2006 POSTER ABSTRACTS", American Journal of Transplantation, 8/2006 <1%

Publication

70 Block, Darci R., and Alicia Algeciras-Schimnich. "Body fluid analysis: Clinical utility and applicability of published studies to guide interpretation of today's laboratory testing in serous fluids", Critical Reviews in Clinical <1%

Laboratory Sciences, 2013.
Publication

---

71 ER, Cenk and OK, Mahmut. "Levels of Cardiac Biomarkers and Coagulation Profiles in ", Kafkas Üniversitesi, 2015. **<1%**
Publication

Exclude quotes	Off	Exclude matches	Off
Exclude bibliography	Off		
